# Solvent-Resistant Lignin-Epoxy Hybrid Nanoparticles for Covalent Surface Modification and High-Strength Particulate Adhesives

**DOI:** 10.1021/acsnano.0c09500

**Published:** 2021-02-17

**Authors:** Tao Zou, Mika Henrikki Sipponen, Alexander Henn, Monika Österberg

**Affiliations:** † Department of Bioproducts and Biosystems, School of Chemical Engineering, Aalto University, Vuorimiehentie 1, 02150 Espoo, Finland; ‡ Department of Materials and Environmental Chemistry, Stockholm University, Svante Arrhenius väg 16C, 10691 Stockholm, Sweden

**Keywords:** hybrid lignin nanoparticle, intraparticle cross-linking, interparticle cross-linking, covalent surface functionalization, lignin-epoxy adhesive, colloidal lignin particle

## Abstract

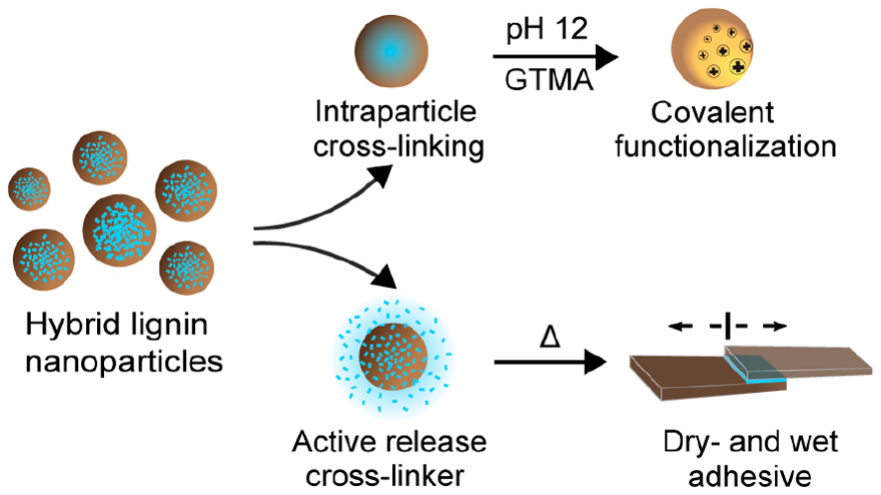

Fabrication of spherical lignin nanoparticles (LNPs) is opening more application opportunities for lignin. However, dissolution of LNPs at a strongly alkaline pH or in common organic solvent systems has prevented their surface functionalization in a dispersion state as well as processing and applications that require maintaining the particle morphology under harsh conditions. Here, we report a simple method to stabilize LNPs through intraparticle cross-linking. Bisphenol A diglycidyl ether (BADGE), a cross-linker that, like lignin, contains substituted benzene rings, is coprecipitated with softwood Kraft lignin to form hybrid LNPs (hy-LNPs). The hy-LNPs with a BADGE content ≤20 wt % could be intraparticle cross-linked in the dispersion state without altering their colloidal stability. Atomic force microscopy and quartz crystal microbalance with dissipation monitoring were used to show that the internally cross-linked particles were resistant to dissolution under strongly alkaline conditions and in acetone-water binary solvent that dissolved unmodified LNPs entirely. We further demonstrated covalent surface functionalization of the internally cross-linked particles at pH 12 through an epoxy ring-opening reaction to obtain particles with pH-switchable surface charge. Moreover, the hy-LNPs with BADGE content ≥30% allowed both inter- and intraparticle cross-linking at >150 °C, which enabled their application as waterborne wood adhesives with competitive dry/wet adhesive strength (5.4/3.5 MPa).

Lignin is a renewable, environmentally friendly, abundant, and low-cost material, which has to date been underexploited due to its structural complexity and heterogeneity.^1−5^ Different strategies have been attempted for lignin valorization for materials applications.^1,5−10^ Among which, lignin-based nanomaterials especially spherical lignin nanoparticles (LNPs) (also called colloidal lignin particles (CLPs) or lignin nanospheres) have emerged in recent years as a research area of broad relevance.^11,12^ Spherical LNPs can be prepared through nanoprecipitation and the resulting hydrodynamic diameter of the particles normally varies between dozens and hundreds of nanometers.^13^ LNPs outperform their crude lignin precursors in the following aspects: (1) LNPs do not aggregate in aqueous media (pH 3–10) due to electrostatic stabilization; (2) LNPs have a large surface area to mass ratio owing to their diameter in the nanoscale; and (3) LNPs have a well-defined spherical shape, and their anionic surface charge allows for physical modifications *via* adsorption of oppositely charged compounds. Those advantages make LNPs attractive building blocks for advanced applications including biomedicine,^14−20^ biocatalysis,^21^ virus removal,^22^ nanocomposites,^9,23^ and Pickering emulsions.^24−27^ However, the dissolution of LNPs at alkaline pH > 10 prevents covalent surface functionalization through industrially relevant chemistries such as epoxy ring-opening reactions.^28−30^ The main reason for this solvent instability is that the LNPs are stabilized at the molecular level by noncovalent forces such as van der Waals, hydrophobic, and π–π interactions.^31−33^ Hence, the solvent stability of LNPs reflects that of the raw material.

The modification of LNPs to tune the surface properties has been mainly achieved by physical adsorption of cationic polymers/oligomers or proteins.^24,27,28,34^ This approach has the drawback of “weak” physical bonding and limited functional group selections. Only a few studies have reported covalently modified LNPs either by attaching the desired functional groups directly to the raw lignin prior to the particle preparation^35^ or by carboxylation of the raw lignin in the solution state followed by amide synthesis in the dispersion state.^36^ Neither of these approaches satisfy the need for resource-efficient and surface-specific functionalization of LNPs.

Stabilization of LNPs by covalent cross-linking appears as a possible solution to their solvent instability. Previous work includes a few examples to this direction. Nypelö *et al*.^37^ cross-linked Kraft lignin with epichlorohydrin *via* a water-in-oil microemulsion template to form intraparticle-cross-linked LNPs that were resistant to dissolution at pH 13. Mattinen *et al*.^38^ used fungal laccases to stabilize LNPs through an enzyme-catalyzed radical-mediated oxidative reaction and obtained tetrahydrofuran-stable LNPs. However, the use of emulsion templates or enzyme-catalyzed cross-linking at markedly low concentrations of LNPs limits the applicability of these approaches.

Here, we show a robust and simple intraparticle cross-linking method that can efficiently stabilize LNPs without altering their surface properties. We employ bisphenol A diglycidyl ether (BADGE) as the cross-linker, because it is hydrophobic, shares a structural resemblance with the aromatic dimers present in softwood Kraft lignin (SKL), and is therefore hypothesized to coprecipitate with SKL to form BADGE-SKL hybrid LNPs (hy-LNPs). We find that the mass ratio of BADGE to SKL is central to the particle morphology, curing behavior, and applicability of the hy-LNPs. We show that the intraparticle-cross-linked hy-LNPs are resistant to dissolution in an acetone-water (3:1, w/w) binary solvent and at pH 12, enabling their chemical functionalization by epoxy ring-opening chemistry. Finally, we demonstrate that the hy-LNP aqueous dispersion can be used as a waterborne adhesive for wood, which outperforms fossil epoxy adhesives both in wet adhesive strength and in expected environmental impacts.

## Results and Discussion

### Preparation and Characterization of BADGE-SKL hy-LNPs

**Figure 1:**
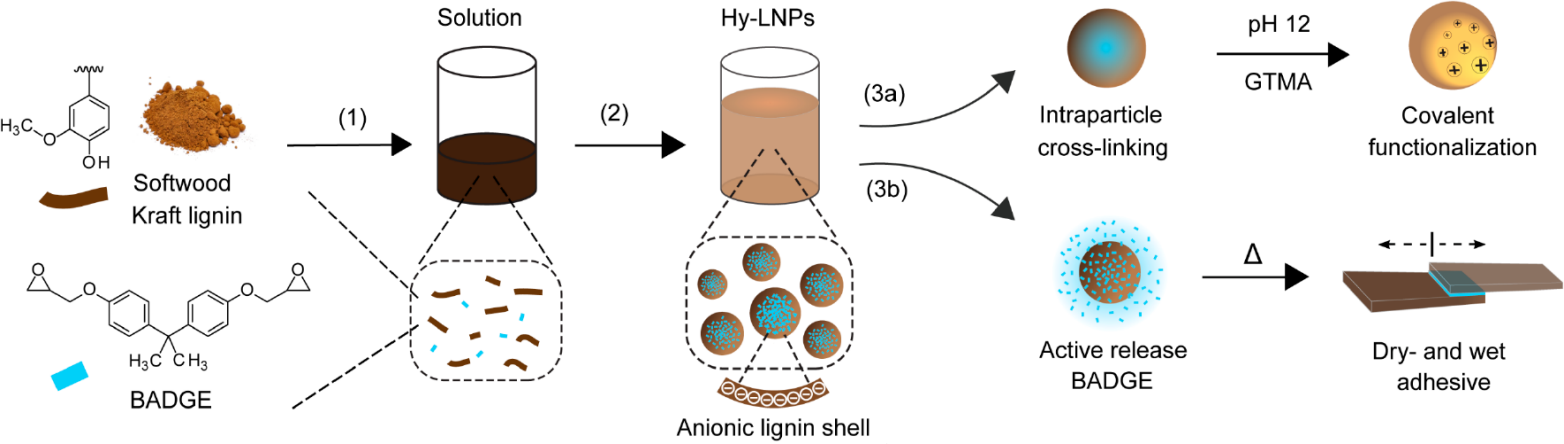
Schematic illustration of the preparation of BADGE-SKL hy-LNPs and their application demonstrations. (1) Codissolution of SKL and BADGE in acetone-water (3:1, w/w). (2) Rapid coprecipitation of SKL and BADGE solution against water to form BADGE-SKL hy-LNPs. (3a) Intraparticle cross-linking of the hy-LNPs (BADGE ≤ 20 wt %) in the dispersion state and covalent surface functionalization of the stabilized particles with glycidyl trimethylammonium chloride (GTMA) at pH 12 *via* epoxy chemistry. (3b) Heat-induced release of BADGE from the hy-LNPs (BADGE ≥30 wt %), allowing both inter- and intraparticle cross-linking for application as a waterborne wood adhesive.

The objective of this study was to structurally stabilize LNPs by intraparticle cross-linking to make them resistant to strong alkaline pH and common organic solvents. To achieve the goal, BADGE-SKL hybrid LNPs (hy-LNPs) were first prepared by coprecipitation (Figure 1). A binary solvent, acetone-water, at a mass ratio of 3:1 was adapted for dissolving SKL and BADGE because this ratio was reported to have the highest solubility for SKL.^39,40^ Five hy-LNP dispersions with BADGE content between 10 and 50 wt % were prepared, and a regular LNP dispersion (0 wt % BADGE) was prepared as a reference. The detailed preparation parameters and final obtained concentrations and yields of the particles are summarized in Table S1.

**Figure 2:**
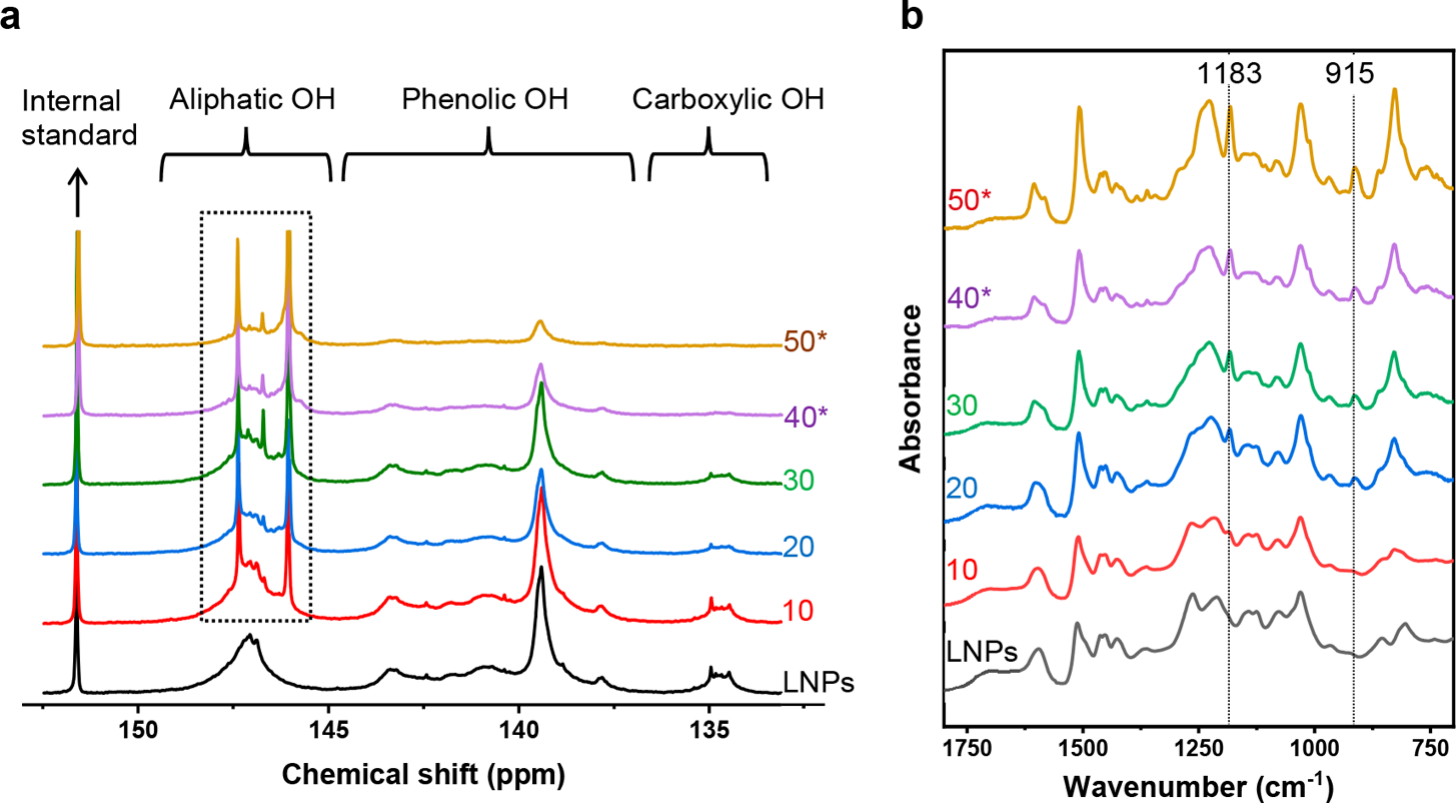
(a) ^31^P NMR spectra of the hy-LNPs (BADGE content: 10–50 wt %) and the regular LNPs. The dashed rectangular box marks the newly formed aliphatic OH peaks (δ = 147.4, 146.7, and 146.0 ppm) occurring to the hy-LNPs due to the ring-opening reaction of oxirane group of BADGE. (b) IR spectra of the hy-LNPs and the regular LNPs. “*” denotes samples where precipitation of the particles occurred in aqueous media during storage at room temperature prior to measurement.

The successful loading and the actual content of BADGE in the hy-LNPs were determined with ^31^P NMR spectroscopy. The newly formed aliphatic OH peaks (δ = 147.4, 146.7, and 146.0 ppm) appearing in the hy-LNPs were assigned to BADGE due to the ring-opening reaction of the oxirane groups, which did not occur in the regular LNPs (Figure 2a). The ring-opening of the oxirane groups was ascribed to the attack by the nucleophilic phosphorylating agent. However, it needs to be emphasized that in case of hy-LNPs40 and hy-LNPs50, carboxylic OH was also involved in the ring-opening reaction, as indicated by the significantly lower ratio of the carboxylic OH to phenolic OH of those particles compared to that of the other hy-LNPs (Table 1). By assuming that the phenolic OH was not involved in the ring-opening reaction at room temperature, the actual content of BADGE could be estimated by comparing the phenolic OH of the hy-LNPs to that of the regular LNPs. The calculated concentrations are summarized in Table 1. The values correlated well with the theoretical ones, except for hy-LNPs50, possibly due to a lower concentration of initially added SKL. ATR-FTIR further showed that the intensities of the distinct absorption bands of BADGE at 1183 cm^–1^ (C–O aromatic ring stretching) and 915 cm^–1^ (C–O stretching of the oxirane group) increased systematically with the increased loading amount of BADGE in the hy-LNPs (Figure 2b).

**Table 1:** Concentrations of Aliphatic, Carboxylic, and Phenolic OH of the hy-LNPs and the Regular LNPs According to Quantitative ^31^P NMR Spectroscopy^a^

sample	aliphatic OH	carboxylic OH	phenolic OH	total OH	wt %^b^
LNPs	2.05	0.44	4.07	6.56	0
hy-LNPs10	2.42	0.35	3.68	6.45	10.5
hy-LNPs20	2.68	0.24	3.38	6.29	17.8
hy-LNPs30	2.34	0.22	2.73	5.28	33.6
hy-LNPs40*	2.29	0.10	2.24	4.63	45.5
hy-LNPs50*	2.43	0.04	1.43	3.91	65.2

a Unit: mmol/g.

b Experimental BADGE content, calculated by (1 – phenolic OH of the hy-LNPs/phenolic OH of the LNPs) × 100%.

**Figure 3:**
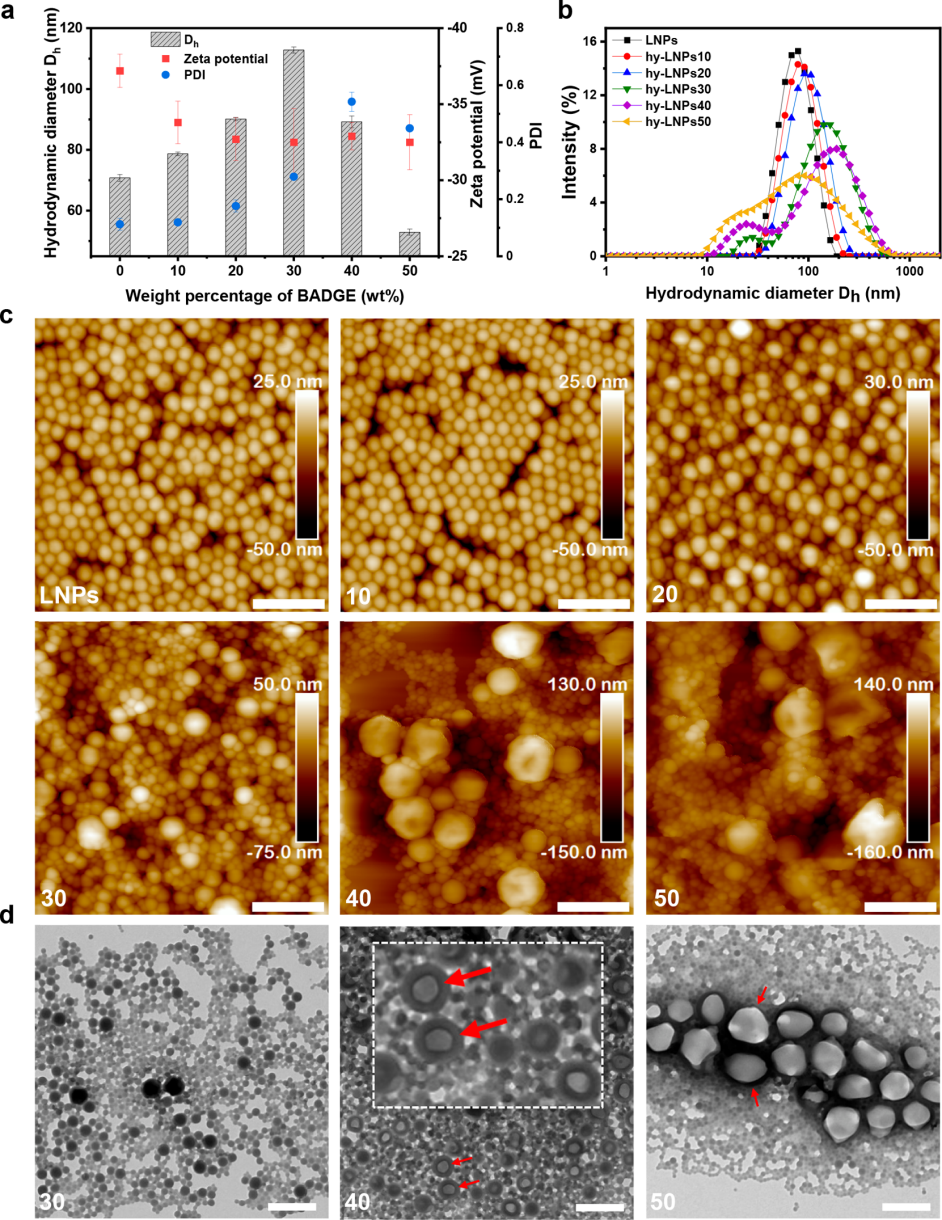
Size distribution, morphology, and ζ potential of the hy-LNPs (BADGE content: 10–50 wt %) and the regular LNPs (0 wt % BADGE). (a) Average hydrodynamic diameters (*D*_h_), ζ potentials, and polydispersity indices (PDI) of the particles. (b) Intensity-based hydrodynamic diameter distributions of the particles. (c) AFM height images of the particles (scale bar: 400 nm). (d) TEM images of the hy-LNPs30, hy-LNPs40, and hy-LNPs50 (scale bar: 400 nm), and selected core–shell structure particles are indicated by the red arrows.

The mass content of BADGE in the hy-LNPs was found to dictate the particle size and size uniformity. The intensity-based average hydrodynamic diameter (*D*_h_) of the particles increased from 71 to 113 nm with the increase of BADGE content from 0 to 30 wt %, then decreased as the BADGE content further increased above 30 wt % (Figure 3a). This decrease was due to the shifting and “expanding” of the small-particle fraction to a smaller diameter as the BADGE content was increased above 30 wt % (Figure 3b). In fact, volume-based particle diameter distributions showed that the small-particle fractions were dominating at the BADGE content ≥30 wt % (Figure S1a). This was further confirmed with atomic force microscope (AFM) and transmission electron microscope (TEM) studies, as shown in Figure 3c,d. Moreover, AFM images also revealed a dented surface of the large particles in hy-LNPs40 and hy-LNPs50 (Figure 3c), suggesting a collapse of those particles upon drying. The large particles with dented surfaces were found to have core–shell structures, as observed with TEM (Figure 3d). Such observations were attributed to the presence of BADGE in liquid form inside the hy-LNPs, which was evidenced by the fact that no melting peaks of BADGE were detected by differential scanning calorimetry (DSC) (see later section). Unlike the particle sizes, the ζ potentials of the different hy-LNPs were close to each other (Figure 3a and Figure S1b), revealing a good stabilization of the noncharged BADGE by the charged SKL. This encapsulation capability of LNPs toward poorly water-soluble compounds is in good agreement with the literature.^16,41^

The colloidal stability of the hy-LNPs in aqueous media was monitored at different temperatures, that is, at room temperature (∼23 °C) and at 4 °C for 110 days and at 80 °C for 24 h. It appeared that the hy-LNPs with a BADGE content ≤20 wt % were stable against precipitation at all temperatures throughout the monitoring period (Figure S2). In contrast, the hy-LNPs with a BADGE content ≥30 wt % precipitated out after a few weeks of storage at room temperature or after a few hours of heating at 80 °C. Combined with the ^31^P NMR results (Figure 2b and Table 1), one can deduce that the precipitation was caused by the reaction of the carboxylic OH of SKL to BADGE that reduced the surface charge of the particles. Nevertheless, these hy-LNP dispersions (BADGE content ≥30 wt %) were stable upon storage at 4 °C for 110 days (Figure S2c), since the oxirane-carboxyl reaction was thermodynamically disfavored at a low temperature. These results suggested that the hy-LNPs with BADGE content ≤20 wt % were suitable for intraparticle cross-linking in dispersion state. In turn, the particles with a BADGE content ≥30 wt % are promising as particulate adhesives since an elevated temperature could extrude BADGE out of the particles to achieve both inter- and intraparticle cross-linking reactions. These two application routes are discussed below.

### Curing of the hy-LNPs in Dispersion State for Surface Functionalization and Wood Adhesives

**Figure 4:**
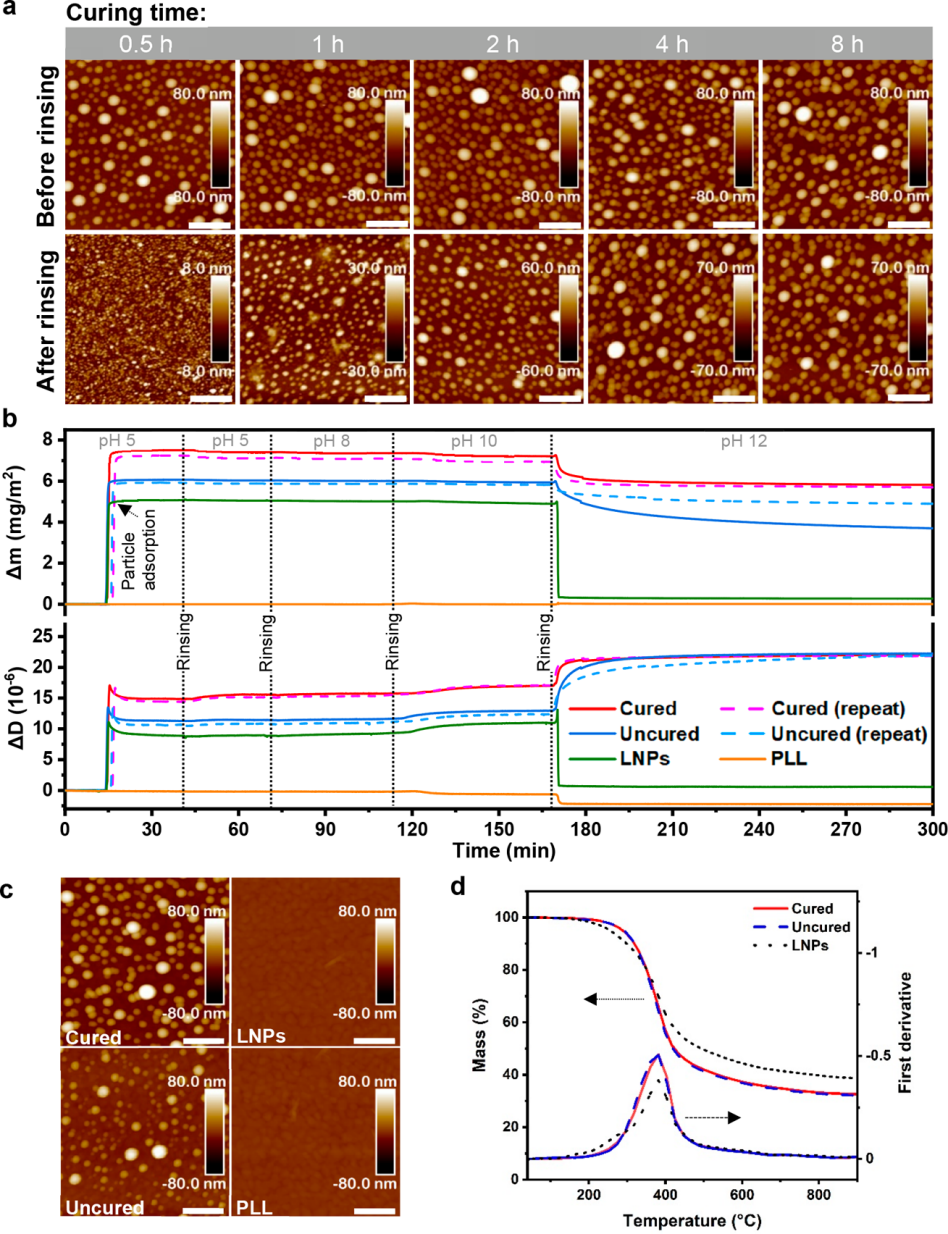
Intraparticle curing of the hy-LNPs20 in dispersion state (∼0.2 wt %) and the resistance of the (4 h) cured particles against dissolution in acetone-water (3:1, w/w) and different pH as well as their thermal stabilities. (a) AFM height images of 0.5–8 h cured hy-LNPs20; samples were measured before and after rinsing with acetone-water (3:1, w/w) (scale bar: 400 nm). (b) QCM-D results of the *in situ* adsorption of the cured and uncured hy-LNPs20 and the regular LNPs, and their response to the pH between 5 and 12. PLL was used as an anchoring polymer for particle adsorption to gold substrates, and hence the response of PLL to pH change was also monitored. (c) AFM height images show particle morphology of the dried samples after QCM-D experiments (*i*.*e*., after treatment at pH 12). The scale bar is 400 nm. (d) Residual mass (%) and first derivative of the residual mass of the dry particles determined with TGA at a heating rate of 10 °C/min.

Screening of the solvent resistance showed that among the intraparticle-cross-linked hy-LNPs with BADGE ≤20 wt %, the particles with 10 wt % BADGE showed partial dissolution (Figure S3), while the ones with 20 wt % of BADGE retained well their particle integrity (Figure 4a). Hence the hy-LNPs20 were used for further investigation. The curing of hy-LNPs20 in dispersion state was performed at their native concentration of ∼0.2 wt % at 105 °C in a sealed glass bottle and was monitored for 8 h. A complete intraparticle cross-linking was achieved within 4 h, as shown by AFM. The 4 h cured particles withheld their integrities after rinsing with acetone-water (3:1, w/w) in contrast to the particles cured for shorter times that showed a clear reduction in size (Figure 4a). Additionally, the cross-linking efficiency of the particles was estimated to be over 60% based on the particle volumes calculated from the average height of the 4 and 8 h cured particles before and after the rinsing with acetone-water (Table S2). The complete reaction of BADGE in the hy-LNPs20 was also revealed by the disappearance of the IR absorption band at 915 cm^–1^ after ≥2 h curing (Figure S4a). The average *D*_h_ of the 4 h cured hy-LNPs20 (∼90 nm) was almost identical to that of the uncured ones (∼90 nm), indicating that the curing did not cause aggregation of the particles (Figure S4b). This outperforms the enzymatic cross-linking of LNPs reported by Mattinen *et al*.,^38^ where a dilute LNP dispersion at 0.01–0.03 wt % was required for conducting intraparticle cross-linking because higher concentrations resulted in interparticle cross-linking and aggregation of the particles. The intraparticle cross-linking was assumed to happen mainly between the phenolic OH of SKL and the oxirane groups of BADGE, as the phenolic OH of SKL (∼4 mmol/g) is much more abundant than the carboxylic OH (∼0.4 mmol/g) (Table 1) and possesses stronger nucleophilicity than the aliphatic OH. A small extent of oxirane-carboxyl reactions occurred as well, as reflected by the minor reduction in absolute ζ potential (Figure S4b) and the DSC results (see later section), which however was insufficient to cause aggregation of the cured particles in aqueous media.

The 4 h cured hy-LNPs20 (hereafter called cured hy-LNPs20) were selected for pH swelling and stability testing using QCM-D and AFM. Uncured hy-LNP20 and regular LNPs were analyzed for comparison. All the particles were stable against dissolution up to pH 10 when subjected to pH-buffered aqueous solutions (Figure 4b). Notably, the adsorbed mass of cured hy-LNPs20 at pH 5 was higher compared to that of uncured hy-LNPs20, probably owing to a slightly lower surface charge density of the cured hy-LNPs20 (Figure S4b). This phenomenon is in line with the AFM results shown in Figure 4a and Table S2. The marked stability of the cured particles was revealed when the pH was increased to 12. The cured hy-LNPs20 displayed only a ∼20% reduction in sensed mass, whereas the regular LNPs showed a sharp decrease in sensed mass back to around zero (Figure 4b). AFM height images confirmed the pH resistance of the cured hy-LNPs20 and a complete dissolution of the regular LNPs at pH 12 (Figure 4c). The dissolution of LNPs was caused by the deprotonation of phenolic OH (p*K*_a_ between 6.2 and 11.3).^42^ The minor mass loss in the case of the cured particles was probably due to partial dissolution of the incompletely cross-linked SKL shell. Interestingly, the initially uncured hy-LNPs20 retained more than 60% of the sensed mass after the alkaline treatment at pH 12, as also revealed by AFM height images (Figure 4b,c). We speculate that the uncured hy-LNPs20 underwent partial intraparticle cross-linking during the increase of the pH since the oxirane-phenol reaction was accelerated with the presence of the base catalyst.^43^ This assumption was also supported by the fact that the uncured hy-LNPs20 remained undissolved at pH 12.9 after the direct addition of 0.1 M sodium hydroxide (Figure S5). Besides, both cured and uncured hy-LNPs20 showed a significant increase in energy dissipation (Δ*D*) at pH 12, indicating softening of the cured LNP20s due to the deprotonation of the phenolic OH. Besides the pH stability, both cured and uncured hy-LNPs20 exhibited a significantly higher *T*_5%_ (5% mass loss) of 290 °C compared to the *T*_5%_ of 256 °C for the regular LNPs (Figure 4d). *T*_5%_ values between 235 and 263 °C have previously been reported for SKL.^44,45^ The identical thermal stability of the cured and uncured hy-LNPs20 originated from the curing of the uncured particles during the heat treatment.

**Figure 5:**
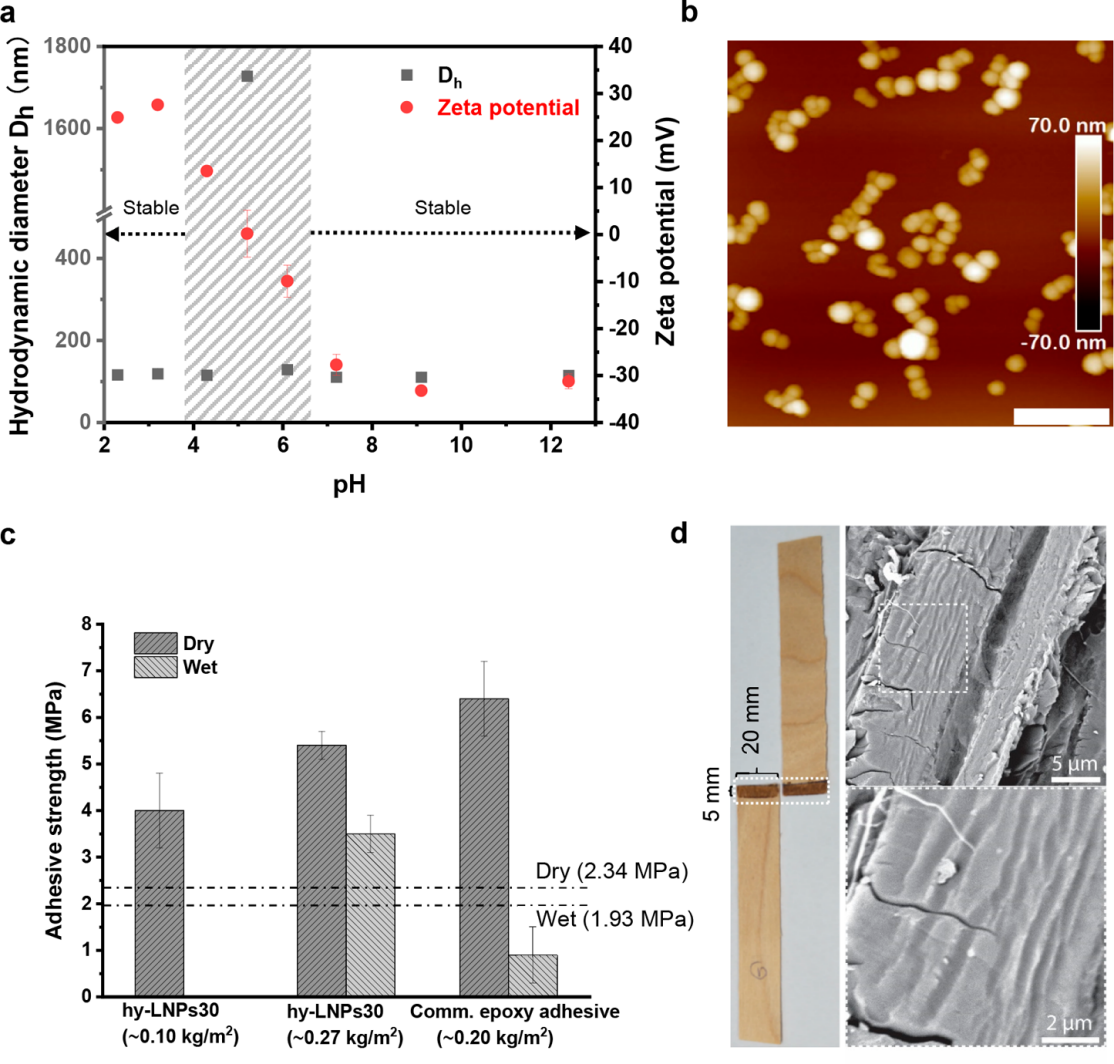
Application demonstration of the hy-LNPs: Covalent cationization of the cured hy-LNPs20 and the adhesive use of the uncured hy-LNPs30 for wood. (a) Average hydrodynamic diameter (*D*_h_) and ζ potential of the cationized cured hy-LNPs20 plotted against pH. The shaded area marks the surface charge transition of the cationized particles. (b) AFM height image shows the particle morphology of the cationized particles obtained at pH 2.3 (scale bar: 400 nm). (c) Adhesive strength of the hy-LNPs30-based waterborne adhesive (41 wt % solid content) and a commercial epoxy adhesive from Loctite for birch veneers (11.5 × 2 × 0.15 cm^3^). Mean ± standard error of three replica are shown. The dashed lines denote the minimum dry/wet adhesive strength requirements for the urea-formaldehyde-type adhesive according to ASTM-D4690. (d) Photographic profile and SEM images of the glued area (20 × 5 mm^2^) after adhesive test.

As a demonstration of the robustness of the cured hy-LNPs20, they were subjected to covalent surface functionalization *via* a base-catalyzed epoxy ring-opening reaction with glycidyl trimethylammonium chloride (GTMA) at pH 12. The resulting cationized particles exhibited a pH switchable surface charge. They were positively charged below pH 4, but negatively charged at pH >6.5 (Figure 5a). From pH 4 to 6.5, the surface charge of the particles underwent a sharp transition from positive to negative, where the particles were in an unstable to metastable state (shaded area in Figure 5a). The transition originated from the deprotonation of the carboxylic OH of SKL that has a p*K*_a_ around 4.^37,38^ A similar pH-responsive charge transition was reported earlier by Sipponen *et al*. for LNPs coated with soluble cationic lignin,^21^ but the covalently functionalized particles presented herein have the advantages of pH stability and ion exchange resistance when subjected to salt solutions. The average *D*_h_ of the covalently cationized particles in the stable regions was close to each other (110–120 nm) regardless of their cationic or anionic net charge. The slightly higher *D*_h_ compared to that of the noncationized ones can be related to a minor aggregation of the cationized particles in aqueous media. In fact, the size of the cationized particles was similar to the noncationized ones, as indicated by AFM height images (Figures 5b and 4a). We envision that beyond the cationization, the intraparticle cross-linked hy-LNPs hold a strong potential for a plethora of other types of chemical functionalization under alkaline conditions, such as the Mannich reaction, and for the applications such as coating, where the particles can resist leaching under harsh environmental conditions. Nevertheless, potential large-scale production of the solvent-resistant hy-LNPs requires evaluation of the cost and toxicity issues arising from BADGE. While a full-scale techno-economic feasibility assessment is out of the scope of this work, we note that the raw material costs would increase from 0.4 to 0.9 €/kg using a market prize of 3 €/kg for BADGE and 0.4 €/kg for SKL.^46^ However, we envision that this approach will still be more economical than the previously reported approaches for obtaining stabilized particles.^37,38^ The toxicity issue of BADGE has been a concern mainly in applications where it is in direct contact with food or beverages, such as inner wall coatings of cans.^47^ In contrast, the ecological effect of BADGE or its possible bisphenol A (BPA) impurities are low because BADGE and BPA biodegrade if released into the environment.^48^ In the current demonstrated application, the BADGE should, furthermore, be fully reacted since the theoretical molar ratio of the phenolic OH of SKL relative to the oxirane groups of BADGE is *ca*. 2:3, that is, an excess of reactive sites of SKL for the preferable first ring-opening reaction of BADGE. In addition, the strong π–π and hydrophobic interactions between BADGE or BPA and SKL favor their associative interactions and prevent leaching of BADGE or BPA into the environment. Nonetheless, a thorough investigation of the leaching of BADGE or BPA and toxicity of the hy-LNPs is needed when using the particles for health-related applications such as biomedicine.

Besides the covalent surface functionalization, the adhesive use of the uncured hy-LNPs was further demonstrated. Among the hy-LNPs with BADGE ≥30%, we selected hy-LNPs30 for adhesive tests because compared to hy-LNPs40 and hy-LNPs50, hy-LNPs30 presented a more uniform size distribution, that is, a better mixture of BADGE and SKL. Moreover, a lower content of BADGE is important for a complete consumption of the epoxy resin. A concentrated hy-LNP30 dispersion (41 wt % solid content) was applied to the birch veneers, and the obtained adhesive results are summarized in Figure 5c. With 0.1 kg/m^2^ loading of the adhesive, the obtained adhesive strength was 4.0 MPa. This value is significantly higher compared to a previously reported value of 2.4 MPa with a formulation of Kraft lignin-glycerol diglycidyl ether-water (1:1:1, w/w/w) under similar curing conditions on plywood but with a higher loading of 0.41–0.46 kg/m^2^.^49^ When we increased the loading concentration to 0.27 kg/m^2^, the adhesive strength further increased to 5.4 MPa under dry conditions and 3.5 MPa in a wet state. Both values significantly surpass the minimum dry/wet adhesive strength requirements for the urea-formaldehyde-type adhesive according to ASTM-D4690. Moreover, the wet adhesive strength of 3.5 MPa was considerably higher than that of the commercial epoxy adhesive used in this study for comparison and the commercial epoxy adhesives reported by Li *et al*.,^49^ indicating a good water resistance of the cured hy-LNPs30. This is a big step forward, as in many cases, epoxy adhesives cannot provide moisture-durable bonds.^50^ The excellent dry and wet adhesive strength of hy-LNP30-based waterborne adhesive for wood is attributed to the molecular level mixture of BADGE and SKL, a thorough inter- and intraparticle cross-linking due to the extrusion of BADGE out of the particles at an elevated temperature, a strong penetration of the adhesives into wood owing to the presence of water, as well as the consumption of the hydrophilic OH groups of SKL during reaction that rendered the adhesive more hydrophobic. The hy-LNPs30 likely formed a layer of molten thermoset after curing, as indicated by the scanning electron microscopic images shown in Figure 5d, whereas the unmodified birch veneer displayed more fragmented morphologies (Figure S6). Note that the failure occurred mainly within the adhesives due to a relatively small gluing area (100 mm^2^) and high thickness (1.5 mm) of the veneers. In fact, failure occurred to the wood when using thin, 0.8 mm-thick veneers (Figure S6). However, these samples could not represent the full adhesive strength of the adhesives. The presence of water in the formulation holds multiple benefits because it (1) enables easy spreading and penetration of the adhesive into wood due to a low viscosity and strong affinity toward wood and (2) reduces the curing temperature and/or time due to the catalytic effect at elevated temperature.^51^ Moreover, the use of unmodified SKL has strong environmental and economic advantages compared to other SKL-based adhesive systems where the prefractionation, degradation, or chemical modification is required, for example, amination of lignin as a hardener for epoxy resin or the solution state epoxidation of degraded/fractionated lignin for preparing lignin-based epoxy resins.^52−55^ Furthermore, the hy-LNP-based waterborne adhesive presented herein is an one-component epoxy adhesive containing no volatile organic compound, which is relatively green and safe compared to formaldehyde-based adhesives that are criticized for their toxicity issues due to emission of formaldehyde during use or the commercial two-component epoxy adhesives that contain toxic hardeners (*e*.*g*., amines). In addition, it is of great economic benefit to replace the commercial epoxy hardener with the low-cost commercial SKL (∼0.4 €/kg^46^). Nonetheless, scaling up production of this formulation requires much more effort, including modification of the production routes, for example, acetone can be removed and recycled by evaporation instead of dialysis to reduce the large use of water. On the other hand, evaporation would require a significant energy input. Nevertheless, the idea of using renewable, environmentally friendly, low-cost SKL as both hardener and stabilizer for water-insoluble resin and release of resin upon heating during use can be the next generation of wood adhesives. Beyond that, this design is also promising to be further expanded for, for example, self-healing coating systems, owing to the nanoencapsulation capacity of SKL toward water-insoluble reagents (*e*.*g*., resin) and release of the reagents upon external stimuli such as heat, pressure, or pH.^56^

### Dry Curing Study of the hy-LNPs to Elucidate the Cross-Linking Mechanism

**Figure 6:**
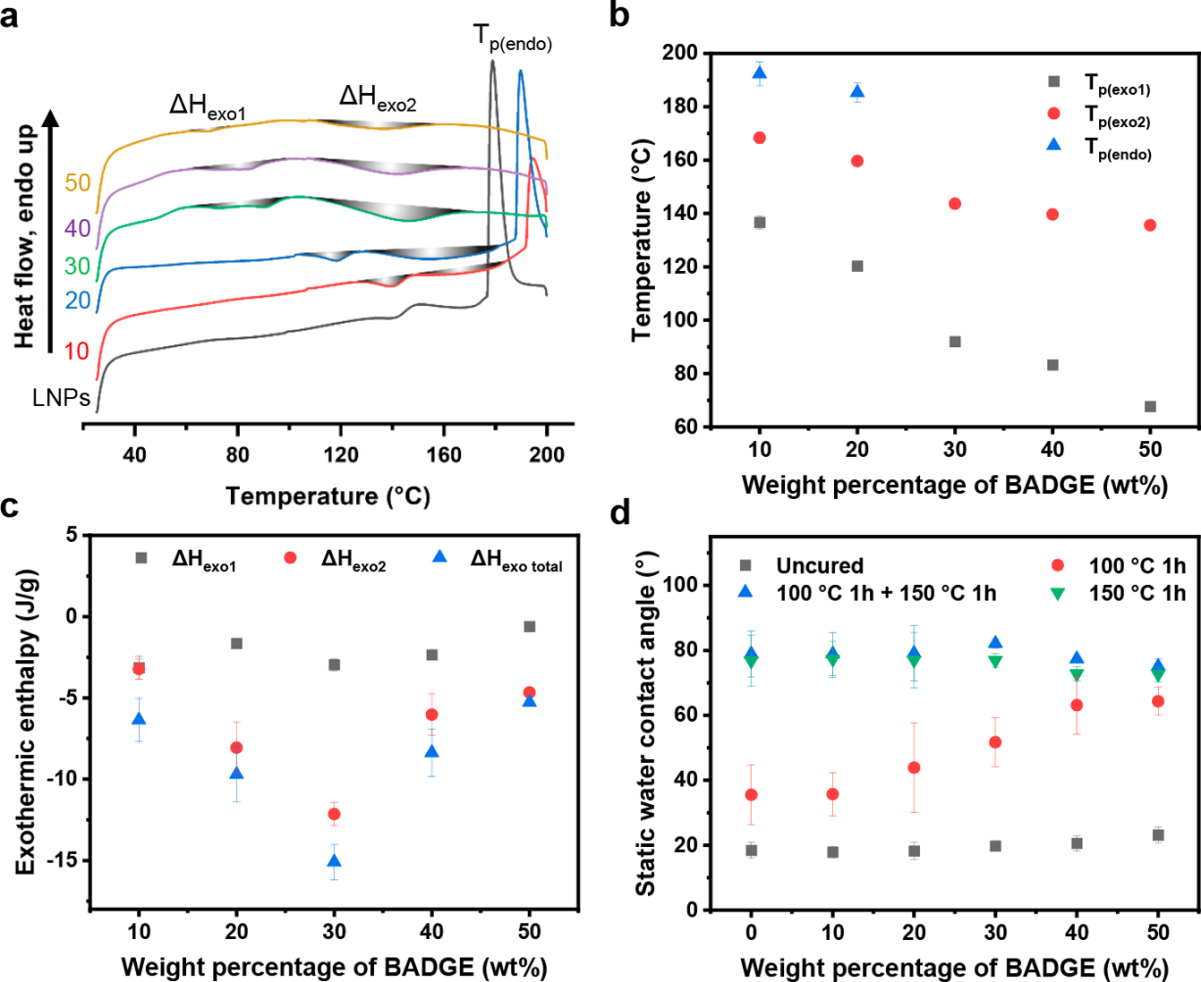
Curing behaviors of the freeze-dried hy-LNPs (BADGE content: 10–50 wt %) and the regular LNPs (0 wt % BADGE) measured with DSC (heating rate: 10 °C/min) and WCA measurements. (a) Typical first heating scans of the particles. Δ*H*_exo1_ and Δ*H*_exo2_ refer to the exothermic enthalpies occurring at a lower and higher temperature, respectively. *T*_p(endo)_ refers to the endothermic peaks observed in LNPs, hy-LNPs10, and hy-LNPs20. (b) Peak temperature of the first exothermic enthalpy (*T*_p(exo1)_), second exothermic enthalpy (*T*_p(exo2)_), and the endothermic enthalpy (*T*_p(endo)_) of the hy-LNPs. (c) First exothermic enthalpy (Δ*H*_exo1_), second exothermic enthalpy (Δ*H*_exo2_), and total exothermic enthalpy (Δ*H*_exo total_) of the hy-LNPs. (d) Static WCAs of the particles after various heat treatments. The particles were treated with heat in three ways: (1) at 100 °C for 1 h, (2) at 100 °C for 1 h, then gradually increasing the temperature to 150 °C and further heating at 150 °C for another hour, and (3) at 150 °C for 1 h. Uncured hybrid particles were measured for comparison. All standard error bars were calculated based on at least three identically prepared samples.

To understand the cross-linking mechanism of the hy-LNPs, the particles were first analyzed with DSC. We found that the hy-LNPs displayed two broad exothermic enthalpy peaks, namely Δ*H*_exo1_ and Δ*H*_exo2_, and Δ*H*_exo2_ dominated the total exothermic enthalpy (Figure 6a). We speculate that Δ*H*_exo1_ and the predominant Δ*H*_exo2_ corresponded to the oxirane-carboxyl and oxirane-phenol reactions, respectively, as carboxylic OH possesses a stronger nucleophilicity than phenolic OH and the concentration of the former is about one-tenth of the latter. Additionally, we observed that the peak curing temperature *T*_p(exo1)_ or *T*_p(exo2)_ decreased linearly as the BADGE content in the hy-LNPs (Figure 6b) increased, probably due to the decrease of the thickness of the SKL shell that allowed a lower reaction temperature. Moreover, we noticed that the hy-LNPs10, hy-LNPs20, and the regular LNPs displayed a sharp endothermic peak at *T*_p(endo)_ > 170 °C (Figure 6a,b) that vanished during the second heating scans (Figure S7). The underlying reason for this thermal behavior that differs from the behavior of the raw SKL (Figure S8) is still unknown and requires further investigation. Besides, it is worth mentioning that the hy-LNPs40 and hy-LNPs50 displayed no melting peaks of BADGE, whereas a sharp melting peak of BADGE occurred at 41 °C for the mechanically blended SKL and BADGE at the mass ratio of 1:1 (Figure S8). Hence, the BADGE molecules likely accumulated as a liquid form in the large particles of hy-LNPs40 and hy-LNPs50, which in turn explained the collapse of those particles as observed in the AFM images shown in Figure 3d. Furthermore, the absolute value of the total exothermic enthalpy |Δ*H*_exo total_| was found to increase with the increase of BADGE content up to 30 wt % and then decrease as the BADGE content further increased (Figure 6c). This phenomenon originated from the incomplete reaction occurring to hy-LNPs40 and hy-LNPs50 during the heating scans, which is discussed in more detail below.

To gain more insights into the curing behaviors of the hy-LNPs, the particles in the form of powders and as monolayers were further analyzed with ATR-FTIR (for powder samples) as well as AFM and contact angle measurements (for monolayer samples). The hy-LNPs were heated in three different ways: (1) at 100 °C for 1 h, (2) at 100 °C for 1 h then gradually increasing the temperature to 150 °C and further heating at 150 °C for another hour, and (3) at 150 °C for 1 h. Heating at 100 °C did not cause observable cross-linking for hy-LNPs10, hy-LNPs20, and the regular LNPs, but a partial cross-linking for hy-LNPs30, hy-LNPs40, and hy-LNPs50, as revealed by ATR-FTIR and AFM measurements (Figures S9–20). This agrees with the *T*_p(exo1)_ determined with DSC (Figure 6b) and the colloidal stability results of the particles (Figure S2). The partial cross-linking was likely related to the oxirane-carboxyl reaction. In contrast to the heating at 100 °C, the other two heating treatments at 150 °C led to cross-linking of all the hy-LNPs. Compared to the heating at 150 °C with a preheating process, a direct heating at 150 °C resulted in a more drastic reaction for the hy-LNPs with a BADGE content ≥30 wt %, especially for hy-LNPs40 and hy-LNPs50. This was indicated by the fact that hy-LNPs40 and hy-LNPs50 formed rigid thermosets after the direct heating, which was not the case for the heating with a preheating process (Figures S17–S20). We believe that the direct heating at 150 °C caused the extrusion of BADGE from the particles for an inter- and intraparticle cross-linking, whereas the preheating process stabilized the particles to some extent and hampered the extrusion of BADGE. This also explains the reduced |Δ*H*_exo total_| when the BADGE content was above 30 wt % (Figure 6c), since the preheating is analogous to the dynamic heating scan in DSC. In terms of hy-LNPs30, partial “melting” of the particles (large ones) occurred after direct heating at 150 °C in contrast to the well-retained particle integrity resulting from the preheating process (Figure S15). However, both heat treatments caused a similar disappearance of the band at 915 cm^–1^ (Figure S16), suggesting a good mixture of BADGE and SKL. As for hy-LNPs10 and hy-LNPs20, the particles withheld their integrity after both heat treatments (Figures S9–S12). Compared to wet curing of hy-LNPs20 at 105 °C, the dry curing at 150 °C appeared less efficient as indicated by the AFM images (Figure 4a and Figure S13), probably because either the curing time (1 h) or temperature (150 °C) was insufficient for dry hy-LNPs20. Besides, a lower curing temperature in the wet state compared to the dry curing is attributed to the catalytic effect of the weakly acidic hot water.^51^

The change in wetting properties of the particles upon different heat treatments supported the property change on the particle surfaces. We found that heating at 100 °C led to the increase of the static water contact angles (WCAs) of the particles from 35 to 70° with the increase of the BADGE content (Figure 6d). This correlates well with the colloidal stability results (Figure S2), manifesting the oxirane-carboxyl reaction on the surfaces especially for hy-LNPs40 and hy-LNPs50. In comparison, the untreated particles all showed similar WCAs at around 20°, suggesting that the hydrophobic BADGE was well-stabilized by the hydrophilic SKL shell. This agrees with the ζ potential results shown in Figure 3a. Previously Qian *et al*.^33^ also reported similar WCAs for the LNPs prepared from acetylated alkali lignin. Surprisingly, either direct heating at 150 °C or heating at 150 °C with a preheating process led to similar increase of the WCAs for all the samples, with resulting WCAs in the range of 75–80° (Figure 6d). Such observations have not been reported in the literature. We speculate that the molecular reorientation of lignin on the particle surfaces played a pivotal role in the increase of hydrophobicity since also the regular LNPs (0 wt % BADGE) showed a similar increase of the WCAs upon the heat treatments. This is indirectly supported by the fact that no chemical variation was detected between the untreated and heat-treated (150 °C, 1 h) LNPs either in bulk or on the particle surfaces, as indicated by ATR-FTIR (Figure S10), ^31^P NMR (Figure S21 and Table S3), and XPS (Figure S22 and Table S4). While compared with other hydroxyl group bearing species, heating-induced wetting property change has been previously reported, for instance, for wood and wax particles.^57,58^

### Hypothesis of hy-LNP Formation Mechanism

It is important to understand the formation of the hy-LNPs to scale up production of these particles in a controlled manner. We suggest that the formation of the hy-LNP using the nanoprecipitation method follows a nucleation–growth process.^16,59,60^ During the nucleation stage, the higher molecular weight SKL molecules supersaturate first and form the critical nuclei due to their lower water solubility and higher hydrophobicity compared to the lower molecular weight ones.^39,40^ BADGE molecules might participate in the formation of the nuclei with and without SKL due to their structural resemblance to SKL, their low water solubility (<0.5 mg/L at 25 °C),^61^ and noncharged nature. Afterward, the nuclei grow into nanoparticles in a random collision and aggregation manner.^59,60^ Lower molecular weight SKL molecules with more carboxylic OH may adsorb onto the nuclei/nanoparticles in parallel with the growth, driven mainly by π–π and hydrophobic interactions,^31−33^ which finally form the shells of the hy-LNPs that stabilize them in the aqueous media.^16^ In this work, an increase in the BADGE content reduced the particle uniformities, probably due to stronger Oswald ripening during the particle formation as BADGE molecules are small, hydrophobic, and noncharged and thus have the tendency to accumulate fast by themselves. While at a lower BADGE content, the Oswald ripening is inhibited since SKL molecules behave as surfactants and prevent the accumulation of BADGE. A strong accumulation of BADGE in the case of higher BADGE content leads to the liquid form of BADGE and thus an observable core–shell structure of the hy-LNPs (*e*.*g*., hy-LNPs40 and hy-LNPs50). While hy-LNPs with lower BADGE content showed no observable core–shell structure (*e*.*g*., hy-LNPs10, hy-LNPs20 and hy-LNPs30), but on the other hand reflect a good compatibility of BADGE and SKL in the particles. In addition, the ζ potential, WCA, and colloidal stability results indicated that all of the hy-LNPs were stabilized by the charged SKL shells. Combining these results with the thermal behaviors of the hy-LNPs, that is, the curing temperature decreased as a function of the increasing BADGE content of the hy-LNPs, suggests that the overall thickness of the SKL shell was thicker in the hy-LNPs with lower BADGE content. A thicker SKL shell is important for colloidal stability of the particles in aqueous media for intraparticle cross-linking. A thinner SKL shell, on the other hand, allows for the extrusion of BADGE from the particles at an elevated temperature to achieve an inter- and intraparticle cross-linking.

## Conclusions

In this work, we systematically studied the coprecipitation of SKL and BADGE with the objective to develop a robust and simple method to stabilize spherical lignin nanoparticles *via* intraparticle cross-linking. Overall, the mass ratio of BADGE to SKL determines the properties of the hy-LNPs in both particle formation and curing steps. With a well-tuned mass ratio of 1:4 of BADGE to SKL, the hy-LNPs can be efficiently structurally stabilized by intraparticle cross-linking while retaining their surface charge and colloidal stability in aqueous media. The internally cross-linked particles exhibited a strong resistance to dissolution at high pH (*e*.*g*., pH 12) and in acetone-water (3:1, w/w) binary solvent as well as improved thermal stability. Covalent cationization was successfully applied to the cross-linked particles *via* epoxy chemistry under strongly alkaline conditions, resulting in a pH-switchable surface charge of the particles. Furthermore, the hy-LNPs with BADGE content ≥30 wt % demonstrated a high potential as a bio-based waterborne adhesive for wood. We envision that such a design of the waterborne adhesive formulation, that is, encapsulation of the water-insoluble epoxy by lignin in aqueous media and release of the epoxy upon heating or applied pressure could trigger opportunities in the controlled release applications and design of many other resin formulations.

## Experimental Section

### Materials

Softwood Kraft lignin (SKL) used in this work was obtained from UPM (Finland) (Trade name: BioPiva 100). The SKL was purified from black liquor using LignoBoost technology. The sugar content in SKL was 0.05 mmol/g, determined with ^13^C NMR in a previous publication.^21^ The number-average molecular weight *M*_n_ and weight-average molecular weight *M*_w_ of SKL were 693 and 4630 g/mol, respectively, determined with GPC (the molar mass distribution and method description can be found in Figure S23). The hydroxy groups of SKL were measured with ^31^P NMR, and the results are shown in Figure 2 and Table 1 in the main text. Bisphenol A diglycidyl ether (BADGE), poly-l-lysine (PLL, 0.1 wt %, *M*_w_ = 150,000–300,000 Da), and glycidyl trimethylammonium chloride (GTMA) were purchased from Sigma-Aldrich. Acetone (100%) was purchased from VWR. All the chemicals were used as received. Deionized (DI) water was used throughout the experiments.

### Preparation of BADGE-SKL hy-LNPs

BADGE-SKL hy-LNPs were prepared by replacing SKL partially with BADGE, but otherwise following the same procedure for preparing LNPs as described earlier.^24^ In brief, SKL and BADGE (total weight of 1 g) with the weight percentage of BADGE varying between 10 and 50 wt % were first codissolved in 100 g of acetone-water (3:1, w/w) under magnetic stirring for 3 h. Undissolved residues were removed by filtering the solutions through paper filters (Whatman, pore size 0.7 μm). Afterward, the solutions were poured rapidly (in seconds) into vortex-stirring DI water (solution: water = 1:2.5, w/w), a process which formed hy-LNPs instantly. Acetone was removed by dialyzing the particle dispersions against DI water using a Spectra/Por 1 tubing with a MWCO of 6–8 kDa. The preparation parameters, final obtained concentrations, and yields of the hy-LNPs are summarized in Table S1.

### Cationization of the Intraparticle-Cross-Linked hy-LNPs

Hy-LNPs20 cured for 4 h at 105 °C in dispersion state were chosen for the covalent cationization reaction. The cationization of the cured particles followed a similar procedure as the cationization of Kraft lignin described in the literature.^62^ In brief, the pH of the cured hy-LNP20 aqueous-dispersion (5 mL) was first tuned to be alkaline (11.7) by adding 0.5 mL of 0.1 M NaOH. Then, 28.1 mg of GTMA was added dropwise to the dispersion. The cationization was conducted at 70 °C for 1 h under stirring. After which, dialysis using a Spectra/Por 1 tubing with a MWCO of 6–8 kDa was applied to the dispersion to remove NaOH and the unreacted GTMA, and the dialysis was continued until the pH reached around 7.

### ^31^P NMR

The hydroxyl groups of the hy-LNPs were quantitatively determined with ^31^P NMR using a Bruker Avance III 400 MHz spectrometer.^63^ The samples were prepared as follows: ∼30 mg (or ∼20 mg for hy-LNPs40 and hy-LNPs50) of the dried particle powders was dissolved in a solvent mixture of 0.8 mL of DMF:pyridine:chloroform-*d*_6_ (0.4:0.6:1, v/v/v) containing the relaxation additive of chromium(III) acetylacetonate (1.63 μmol) and the internal standard of *N*-hydroxy-5-norbornene-2,3-dicarboxylic acid imine (10 μmol). The hydroxy groups were phosphitylated with 0.15 mL of 2-chloro-4,4,5,5-tetramethyl-1,3,2-dioxaphospholane (Sigma-Aldrich). A total of 128 scans with 1 s acquisition time and 5 s pulse delay (zgig with 90° pulse angle) were recorded for the analysis of data.

### ATR-FTIR

The infrared (IR) absorbance of the samples were measured with an attenuated total reflection - Fourier transform infrared spectroscopy (ATR-FTIR) (PerkinElmer, Spectrum Two FT-IR Spectrometer). The aqueous particle dispersions were freeze-dried prior to the measurement. A total of 4 scans with a resolution of 1 or 0.25 cm^–1^ were used for sample measurement.

### Hydrodynamic Diameter and ζ Potential Analysis

The hydrodynamic diameter *D*_h_ and ζ potential of the particles were analyzed using a Zetasizer Nano ZS90 instrument (Malvern Instruments Ltd., U.K.). The refractive index (RI) and viscosity of the dispersant (water) were set to be 1.33 and 0.8872 cP respectively. The RI and absorption of the particles were set to be 1.4 and 0.9, respectively. The average hydrodynamic diameter *D*_h_ was determined by the system based on the scattered light intensities (scattering angle of 90°). The ζ potential was determined with a dip cell probe using automatic voltage, and the Helmholtz–Smoluchowski equation^64^ was adapted for obtaining the results. The *D*_h_ of the hy-LNPs and the regular LNPs were measured at their original concentrations (∼0.2 wt %), and the ζ potentials were measured at a diluted concentration (∼0.02 wt %, diluted with DI water, pH varied from 5 to 5.6 after dilution). *D*_h_ and ζ potential of the cationized particles were measured at a concentration of ∼0.1 wt %. Average values of three replicates of the *D*_h_ and ζ potentials were used in the analysis and reporting of data.

### AFM

A MultiMode 8 atomic force microscope (AFM) equipped with a NanoScope V controller (Bruker Corporation, U.S.A.) was used to analyze the samples. All the images were obtained in tapping mode in ambient air using NCHV-A tapping mode probes (Bruker). The samples were prepared in two different ways: (1) dropping of 5 μL of the diluted aqueous particle dispersion (diluted to be 0.02 wt % by DI water) on a mica surface followed by ambient drying; and (2) direct adsorption of the particles onto PLL-modified silicon wafer by immersing the wafer (∼1 × 1 cm^2^) in the particle dispersion (at the native concentration) for 1 h, followed by rinsing with DI water and N_2_ drying. A silicon wafer purified with 15 min UV/ozone treatment in an Ozonator was modified with PLL by immersing the wafer in PLL solution for 1 h, followed by rinsing with DI water and N_2_ drying before applying the particles. Nanoscope Analysis (version 1.5, Bruker) was used for image processing.

### TEM

Transmission electron microscopic (TEM) images of the hy-LNPs were obtained in bright-field mode on a FEI Tecnai 12 (USA) operating at 120 kV. The samples were prepared by dropping 5 μL of the diluted particle aqueous dispersion (diluted to be 0.02 wt % by DI water) on a carbon film support grid, followed by incubating (1 min) and blotting of the excess water with a filter paper. The samples were further dried overnight at ambient conditions prior to TEM measurements.

### SEM

A Phenom scanning electron microscopy (Phenom Pure G5) with a resolution of 30 nm was used to analyze the cured hy-LNPs30 on birch veneer surfaces. The samples were coated with gold–palladium (Au80Pd20) using a Q 150R S plus rotary pumped coater (20 mA, 30 s) prior to SEM analysis.

### QCM-D

*In situ* adsorption of the particles along with the pH swelling and stability of the particles were performed on a QCM-D E4 (Q-Sense, Sweden) in continuous flow mode. Gold QCM crystals (Q-sense, Sweden) modified with PLL (following the same protocol as for the modification of silicon wafers for AFM measurements) were used as the substrates. The flow rate of 100 μL/min and the temperature of 25 °C were used throughout the measurements. After obtaining a stable baseline with pH 5 solution, the diluted aqueous particle dispersions (diluted to be 0.05 wt % by DI water, pH around 5) were pumped into the QCM-D for *in situ* adsorption. After that, pH 5, 8, 10, and 12 solutions were pumped stepwise to elucidate the pH swelling and stability of the particles. The different pH solutions were prepared by adding HCl or NaOH to DI water. The change of mass (sensed mass) was calculated according to Sauerbrey equation,^65^ as follows:
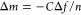
where Δ*m* is the change of mass, Δ*f* is the change of resonance frequency, *C* is a constant (0.177 mg/m^2^ Hz) that describes the sensitivity of the device, *n* is the overtone number (*n* = 1, 3, 5, 7, 9, 11; *n* = 1 represents the fundamental frequency at 4.95 MHz). The dissipation factor *D* is used to indicate the viscoelasticity of the adsorbed layer, which is defined as
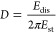
where *E*_dis_ and *E*_st_ denote the dissipated and stored energy, respectively, during one oscillation cycle. The dissipation change is calculated as, Δ*D* = *D* – *D*_0_, where *D*_0_ is the baseline dissipation. In this work, the fifth overtone of frequency change (Δ*f*_5_) and dissipation change (Δ*D*_5_) were used for calculation and reporting of data.

### DSC

The curing behavior of the particles were analyzed with differential scanning calorimetry (DSC) (Mettler Toledo DSC 3+). The freeze-dried particle powders were prepared for DSC measurements. The powders were loaded in 40 μL aluminum pans; the loaded amount varied between 5 and 11 mg. The samples were heated from 25 to 200 °C, cooled down to 25 °C, and then heated up to 200 °C again. The heating/cooling rate was at 10 °C/min. Mass loss of the samples was observed after DSC measurements ascribing to the evaporation of residual water. The mass loss was <0.2% for hy-LNPs30, hy-LNPs40, and hy-LNPs50, 1–3% for hy-LNPs10 and hy-LNPs20, and 3–4% for LNPs. The average values of three identically prepared samples were used for the report of data.

### TGA

The thermal stability of the particles was analyzed with thermogravimetric analysis (TGA) (Netzsch STA 449 F3 Jupiter & QMS 403 Aëolos Quadro). The freeze-dried particle powders were loaded in 85 μL aluminum pans; the loaded amount varied between 7 and 11 mg. The samples were heated from 40 to 900 °C at a heating rate of 10 °C/min in the atmosphere of helium gas (50 mL/min).

### Adhesive Strength Analysis

A concentrated hy-LNPs30 aqueous dispersion (∼41 wt % solid content) obtained from the sediment after centrifugation (11000 rpm for 30 min) was used for the adhesive analysis. Birch veneers with the size of 11.5 × 2 × 0.15 cm^3^ were loaded with the hy-LNPs30 dispersion over an area of 1 cm^2^ using two different loading concentrations (∼0.10 and ∼0.27 kg/m^2^). Then the veneers were paired and hot-pressed at 160 °C and 0.7 MPa for 10 min to prepare the samples for adhesive strength test. A commercial multipurpose epoxy adhesive comprising of an epoxy resin and a hardener purchased from Loctite was used as reference. After applying ∼0.20 kg/m^2^ of the commercial epoxy adhesive to the veneers, the veneers were pressed at 0.7 MPa for 20 min and then allowed to be cured for 24 h at room temperature. The adhesive strength analysis was performed on an automated bonding evaluation system (Adhesive Evaluation Systems Inc., United States). The wet adhesive strength was measured after soaking the cured veneers in deionized water (at room temperature) for 48 h. Three identically prepared samples were measured.

### Contact Angle Measurements

The static WCAs of a monolayer of the particles were measured with a KSV CAM 2000 (KSV Instruments Ltd., Finland). The size of the water droplet was controlled to be around 6.0 μL. A video camera was used to record 40 images at 1 s intervals, and the last image was used for WCA calculation. The samples were prepared by direct adsorption of the particles onto PLL-modified silicon water as descried earlier. At least three identically prepared samples were used for analysis and report of data. As a background reference, the WCA of PLL-modified silicon wafer was measured to be 48.6 ± 3.7°.

## Supporting Information Available

Table showing the preparation parameters, final obtained concentrations, and yields of the hy-LNPs and LNPs; volume-based hydrodynamic diameter distributions and ζ potential distributions of the hy-LNPs and LNPs; colloidal stability of the hy-LNPs in aqueous media at various temperatures; vuring of hy-LNPs10 in dispersion state and their resistance to acetone-water (3:1, w/w) after curing; table showing the average particle heights of 0.5–8 h cured hy-LNPs20, before and after rinsing with acetone-water (3:1, w/w); IR absorption of 0 to 8 h cured hy-LNPs20; *D*_h_ and ζ potential of the uncured and 4 h cured hy-LNPs20; DLS results of the uncured hy-LNPs20 at pH 12.9; scanning electron microscopic images of the veneer surfaces with and without cured hy-LNPs30; photo showing the wood failure occurring to the 0.8 mm-thick veneers during adhesive strength test; DSC results of the hy-LNPs, mechanically blended SKL and BADGE mixture, BADGE (in solid form) and SKL; AFM and ATR-FTIR results of the hy-LNPs and LNPs after various heat-treatments; ^31^P NMR and XPS results of the untreated and heat-treated LNPs; SKL molar mass distribution determined with GPC (PDF)
