# Supplementary material for: Solvent-Resistant Lignin-Epoxy Hybrid Nanoparticles for Covalent Surface Modification and High-Strength Particulate Adhesives
Source: ACS Nano. 2021 Feb 17;15(3):4811–23. doi: 10.1021/acsnano.0c09500 (PMC8023795; doi:10.1021/acsnano.0c09500)
Supplement: Supplementary file 1 — nn0c09500_si_001.pdf [file nn0c09500_si_001.pdf]

Supporting information for

Solvent-Resistant Lignin-Epoxy Hybrid Nanoparticles for Covalent Surface Modification and High-Strength Particulate Adhesives

Tao Zou[†], *Mika Henrikki Sipponen*[‡], *Alexander Henn*[†] *and Monika Österberg*[†, *]

[†]Department of Bioproducts and Biosystems, School of Chemical Engineering, Aalto University, Vuorimiehentie 1, 02150 Espoo, Finland

[‡]Department of Materials and Environmental Chemistry, Stockholm University, Svante Arrhenius väg 16C, 10691, Stockholm, Sweden

*Corresponding author: monika.osterberg@aalto.fi

KEYWORDS: hybrid lignin nanoparticle, intraparticle-crosslinking, interparticle-crosslinking, covalent surface functionalization, lignin-epoxy adhesive, colloidal lignin particle

Table S1. Preparation parameters, final obtained concentrations and yields of the BADGE-SKL hybrid LNPs and the regular LNPs.

Sample code	Weight percentage of BADGE relative to SKL (wt %)	Initial total concentration of BADGE and SKL solution (wt %)	Final obtained concentration of the particles in water (wt %)	Yield (wt %)
LNPs	0	1	0.23	85.3
hy-LNPs10	10	1	0.19	78.8
hy-LNPs20	20	1	0.21 ± 0.01[a]	79.3 ± 2.2[a]
hy-LNPs30	30	1	0.20 ± 0.01[a]	72.5 ± 3.7[a]
hy-LNPs40	40	1	0.19 ± 0.01[a]	70.1 ± 3.0[a]
hy-LNPs50	50	1	0.19 ± 0.02[a]	69.8 ± 0.7[a]

[a] Mean value ± absolute deviation of two batches. The pH values of all the final obtained aqueous dispersions were between 4 and 5.

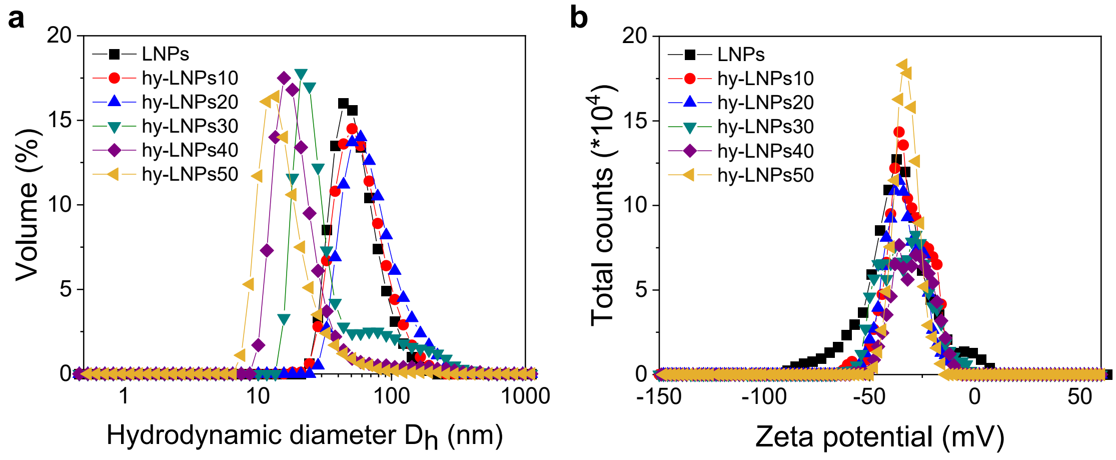

Figure S1. (a) Volume-based hydrodynamic diameter distributions of the hy-LNPs and the regular LNPs. (b) Zeta-potential distributions of the hy-LNPs and the regular LNPs.

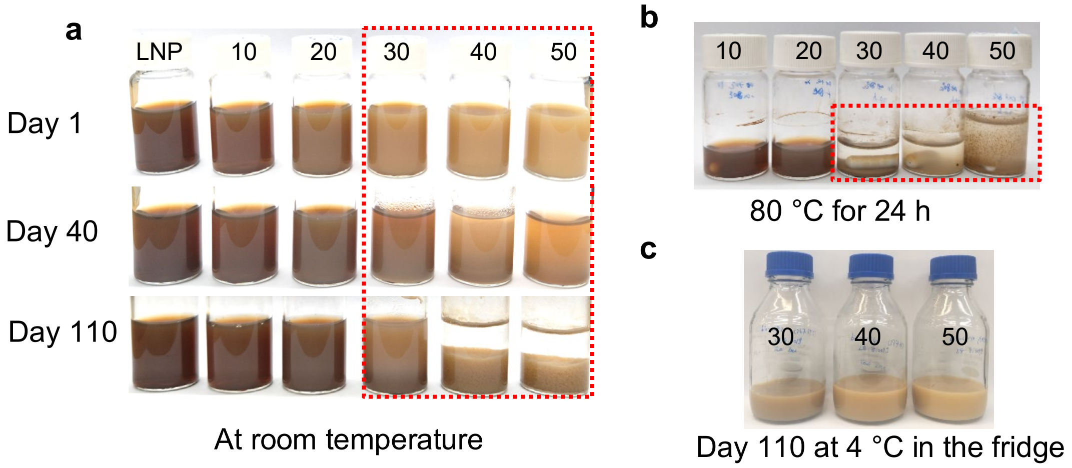

Figure S2. Colloidal stability of the hy-LNPs (10 to 50 wt% BADGE) and the regular LNPs (0 wt% BADGE) in aqueous media at different temperatures. (a) At room temperature (~23 ºC) for 110 days, the dashed red rectangular box marks the gradual precipitation of hy-LNPs30, hy-LNPs40 and hy-LNPs50. (b) After heating at 80 °C for 24 hours, the dashed red rectangular box marks the precipitation of hy-LNPs30, hy-LNPs40 and hy-LNPs50. (c) After 110 days at 4 °C for hy-LNPs30, hy-LNPs40 and hy-LNPs50, no precipitation was observed.

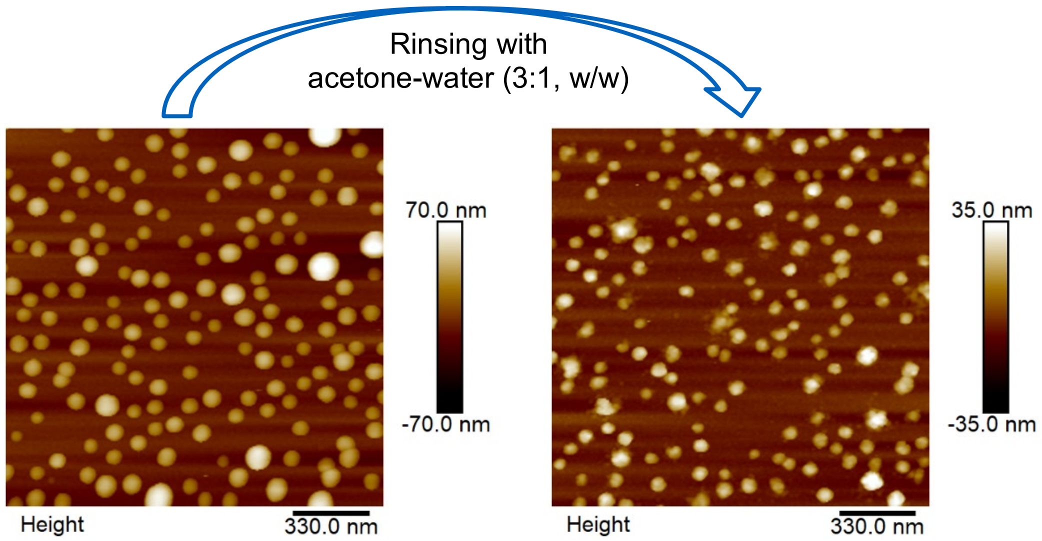

Figure S3. AFM height images of the 24 h-cured hy-LNPs10 (in dispersion state at 105 °C) before and after rinsing with acetone-water (3:1, w/w).

Table S2. Average particle heights of 0.5 to 8 hour cured hy-LNPs20 (at 105 °C in dispersion state) before and after ring with acetone-water (3 : 1, w/w), calculated based on the AFM-height images shown in Figure 4a in the main article (Unit: nm). The average height was calculated using Particle Analysis in Nanoscope 1.5, with the principle that the counted particle numbers were at maximum in each image (with the least over 180 counts). Images were processed with plane fit (3rd) prior to Particle Analysis.

Curing time	0.5 h	1 h	2 h	4 h	8 h
Ave. height (before)	32.8 ± 15.1	34.9 ± 15.9	32 ± 15.3	43.5 ± 14.4	46.0 ± 14.4
Ave. height (after)	n.a.	15.7 ± 7.5	27.8 ± 11.1	39.4 ± 13.0	36.2 ± 11.7

The higher average heights of 4 and 8 hours-cured particles (before rinsing) compared to the particles cured with less hours can be attributed to slight aggregation of the particles. The slight aggregation was due to small extent of carboxyl-oxirane reaction that reduced surface charge density of the particles (Figure S4b).

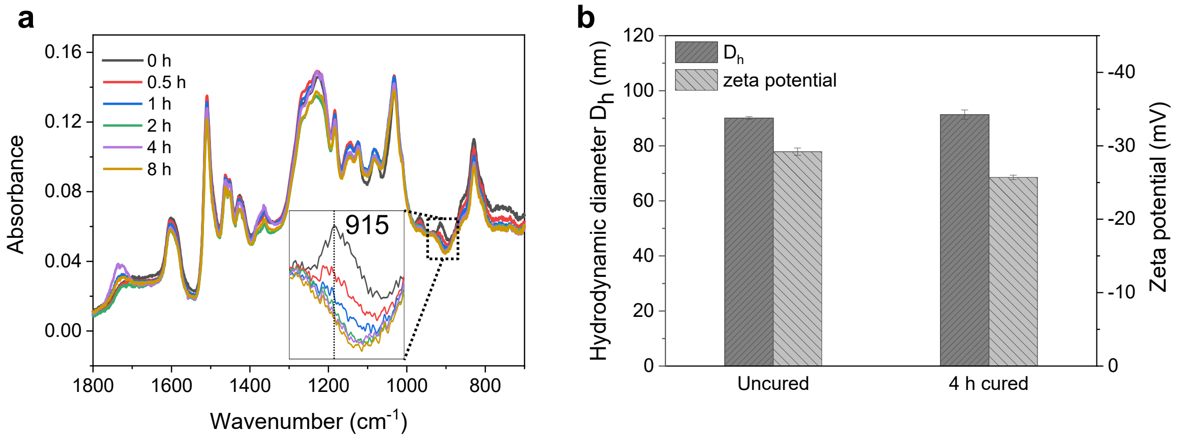

Figure S4. (a) IR absorbance of 0 to 8 hour cured hy-LNPs20 (the absorbance differences at the wavenumber around 1750 cm^{-1} was likely caused by the residue acetone that was used to clean the diamond surface). (b) Average hydrodynamic diameter (D_h) and zeta potential of the uncured and 4 h cured hy-LNPs20.

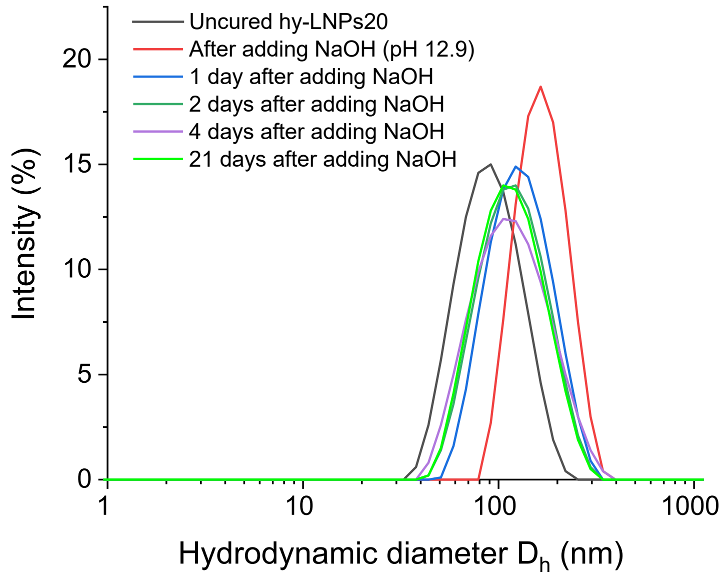

Figure S5. Dynamic light scattering results of the uncured hy-LNPs20 in aqueous media after direct addition of 0.1 M sodium hydroxide (pH 12.9), monitored for 21 days.

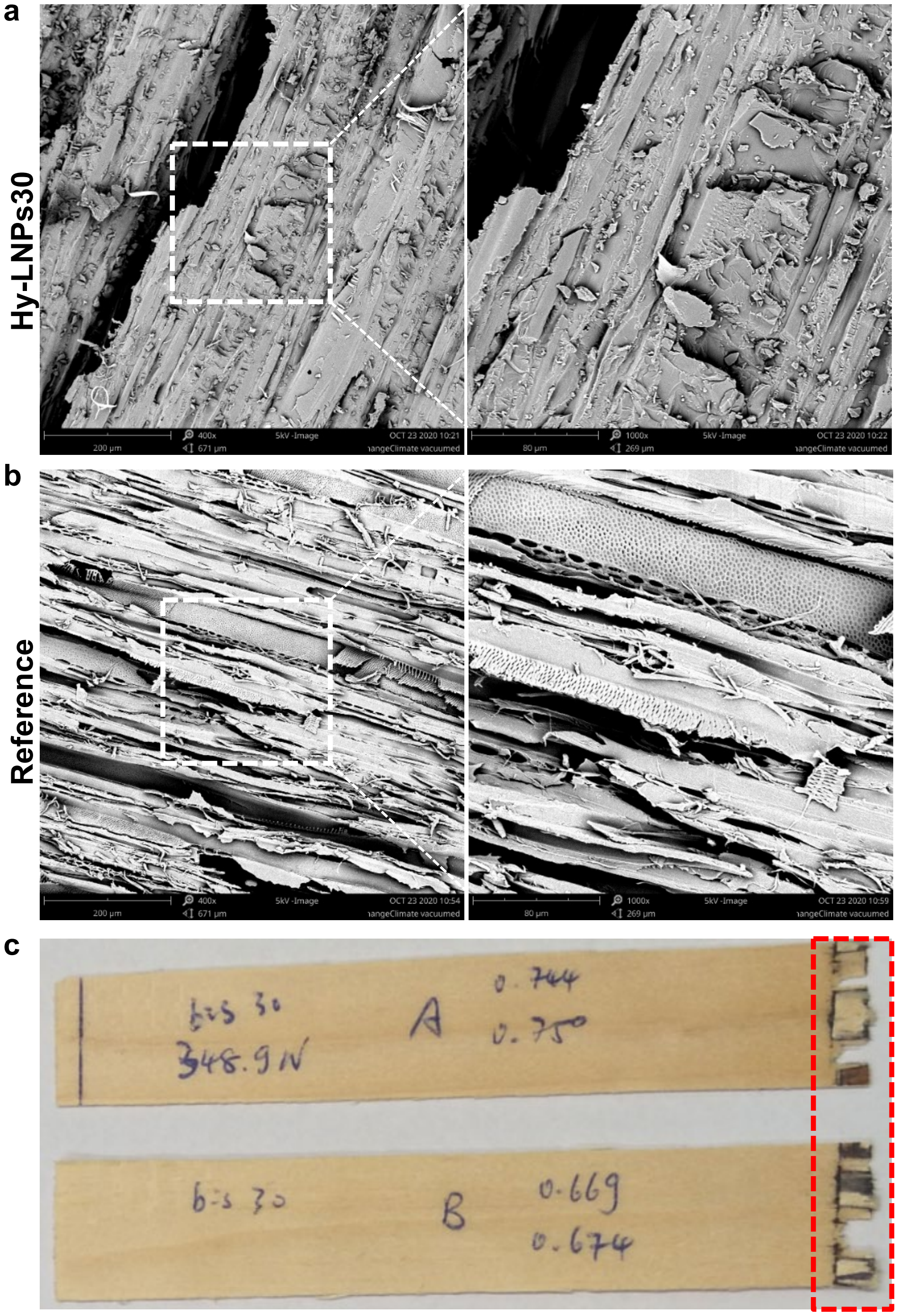

Figure S6. (a) Scanning electron microscopic images of the cured hy-LNPs30 on the birch veneer surface after adhesive strength test. (b) Scanning electron microscopic images of the unmodified veneer surface as the reference. (c) Picture showing the wood failure (dashed red box marked area) occurring to the 0.8 mm-thick veneers during the adhesive strength test. The loading concentration of hy-LNP30 dispersion was 0.11 kg/m^2 and the resulting adhesive strength was 3.5 MPa.

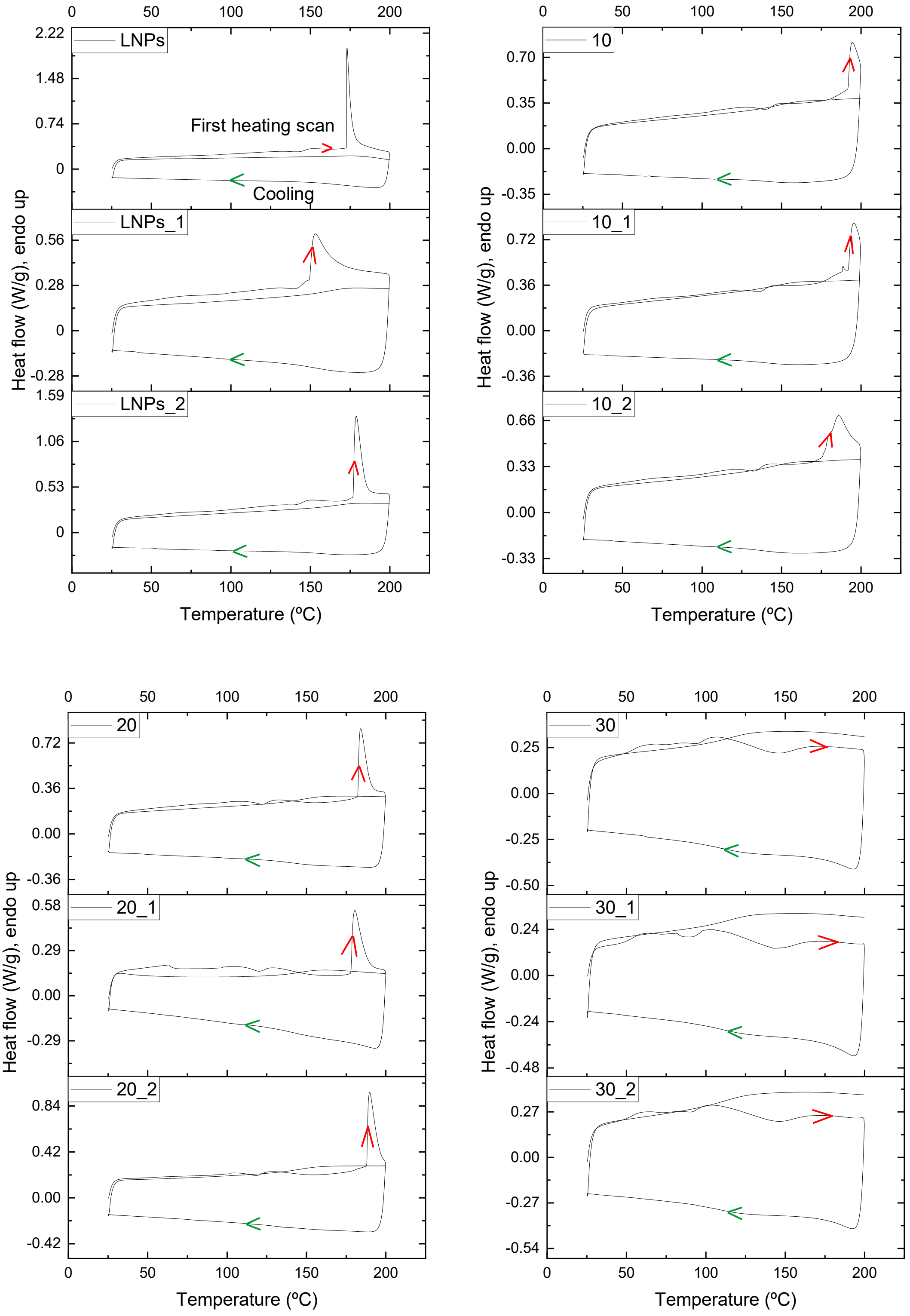
LNPs
First heating scan
Cooling
LNPs_1
LNPs_2
10
10_1
10_2
20
20_1
20_2
30
30_1
30_2
Heat flow (W/g), endo up
Temperature (ºC)

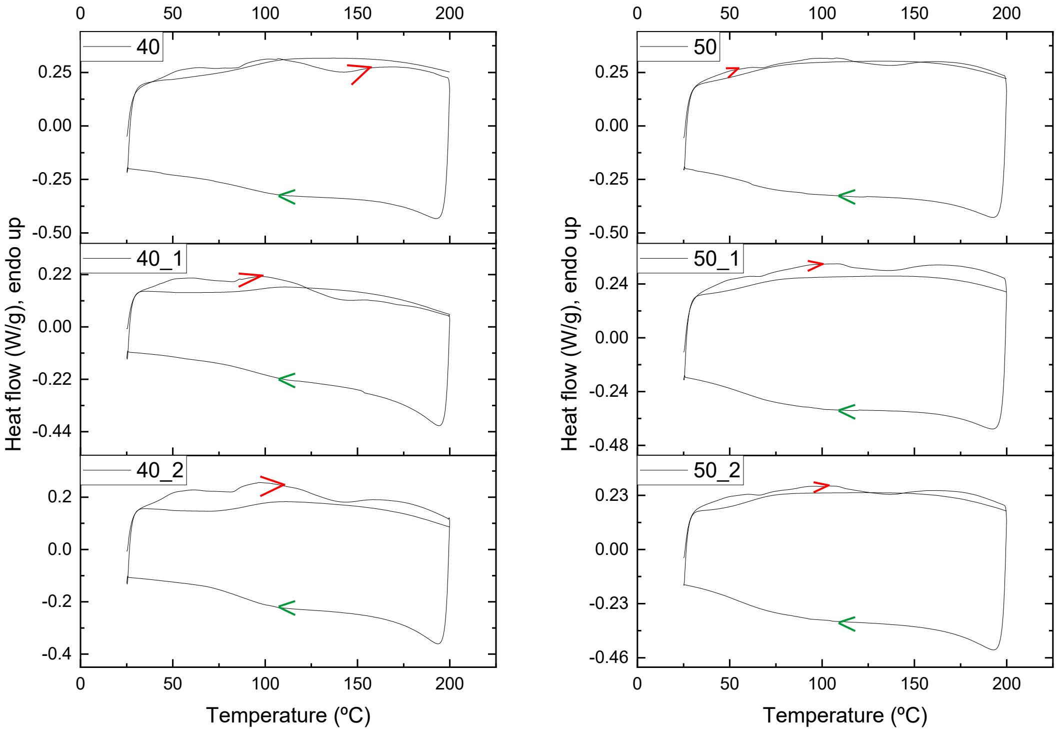

Figure S7. Curing behaviors of the hy-LNPs (10 to 50 wt% BADGE) and the regular LNPs measured with DSC. The samples were heated from 25 to 200 °C (red arrow), cooled down to 25 ºC (green arrow), then heated up to 200 ºC again. Heating/cooling rate: 10 ºC/min. Three identically prepared samples were measured.

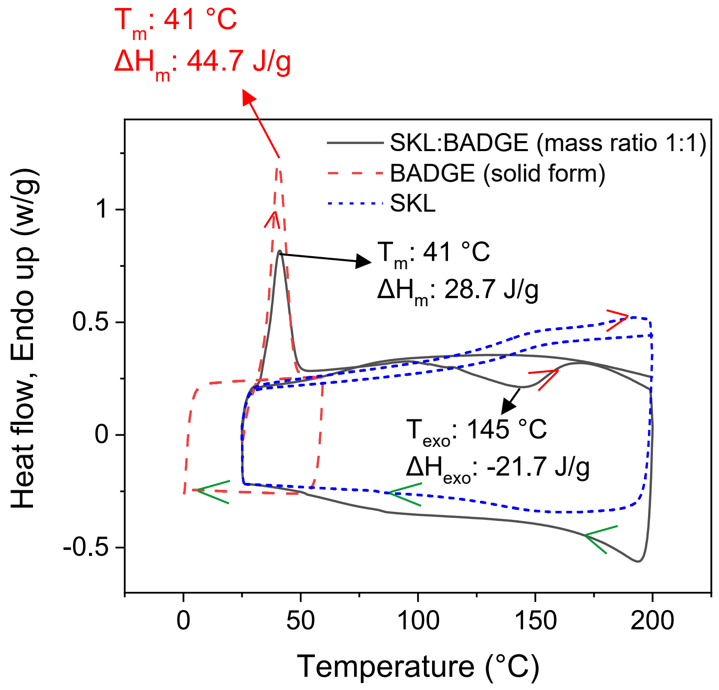

Figure S8. Curing behaviors of mechanically blended SKL-BADGE mixture (mass ratio: 1 to 1), BADGE (in solid form) and SKL. SKL-BADGE mixture and SKL were heated from 25 to 200 °C, cooled down to 25 °C, then heated up to 200 °C again. Solid BADGE was heated from 0 to 60°C, cooled down to 0 °C, then heated up to 60 °C (no melting peak was observed during the second heating scan). Heating/cooling rate: 10 °C/min. Red and green arrows mark the first heating scans and the cooling scans respectively.

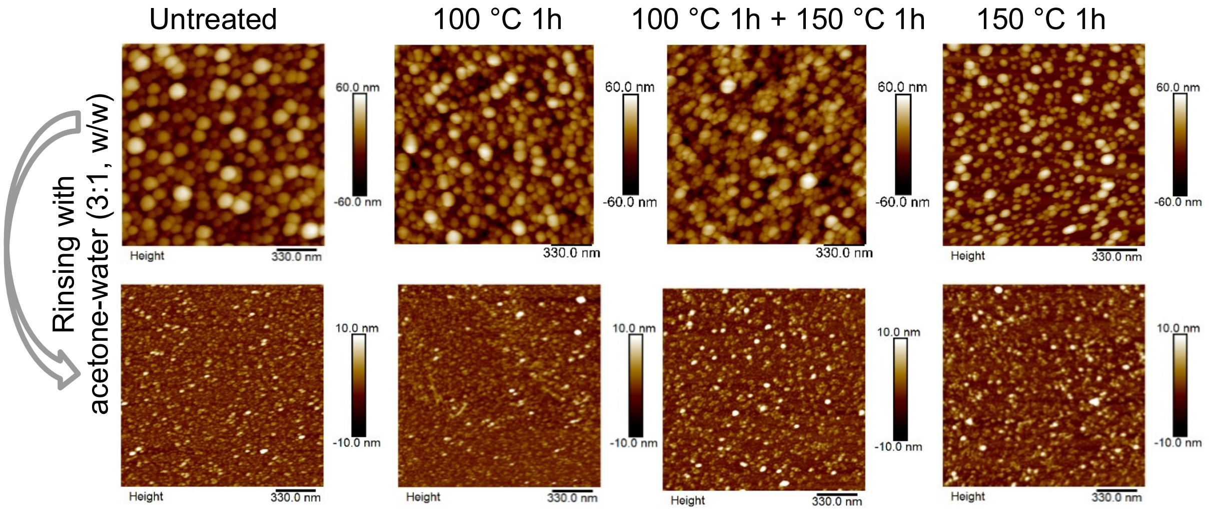

Figure S9. AFM height images of different heat-treated dry LNP monolayer, before and after rinsing with acetone-water (3:1, w/w) are shown. Heat-treatment: 1) at 100 °C for 1 hour; 2) at 100 °C for 1 hour, then gradually increasing the temperature to 150 °C and further heating at 150 °C for another hour; 3) at 150 °C for 1 hour.

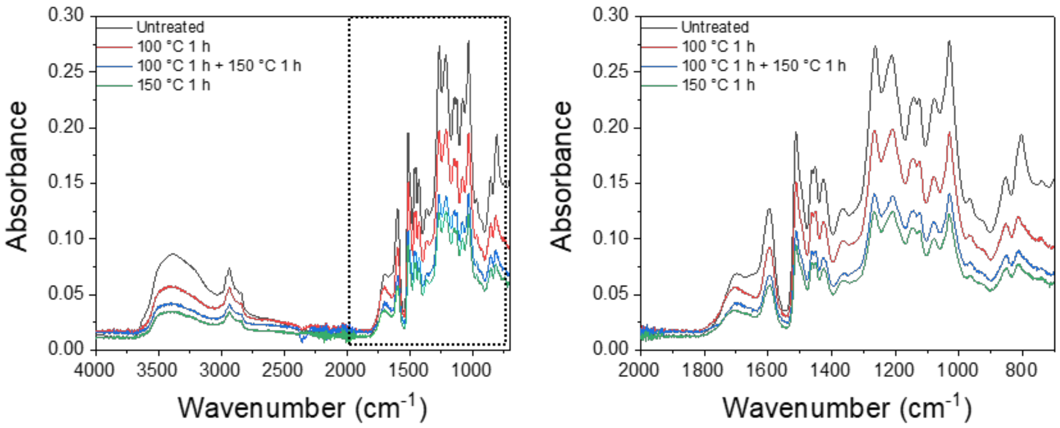

Figure S10. ATR-FTIR spectra of different heat-treated dry LNP powders.

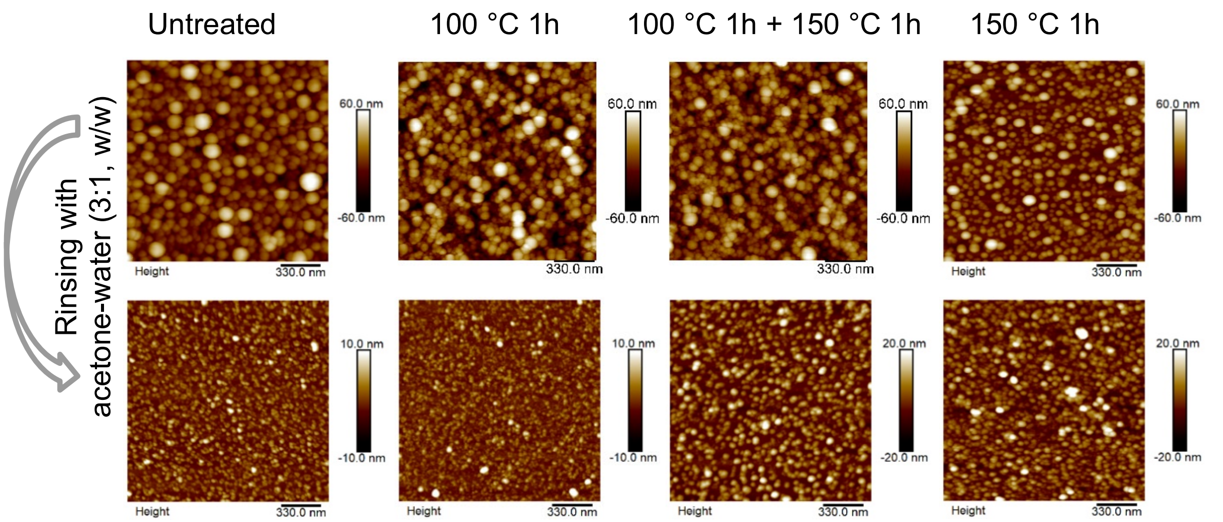

Figure S11. AFM height images of different heat-treated dry hy-LNP10 monolayer, before and after rinsing with acetone-water (3:1, w/w) are shown. Heat-treatment: 1) at 100 °C for 1 hour; 2) at 100 °C for 1 hour, then gradually increasing the temperature to 150 °C and further heating at 150 °C for another hour; 3) at 150 °C for 1 hour.

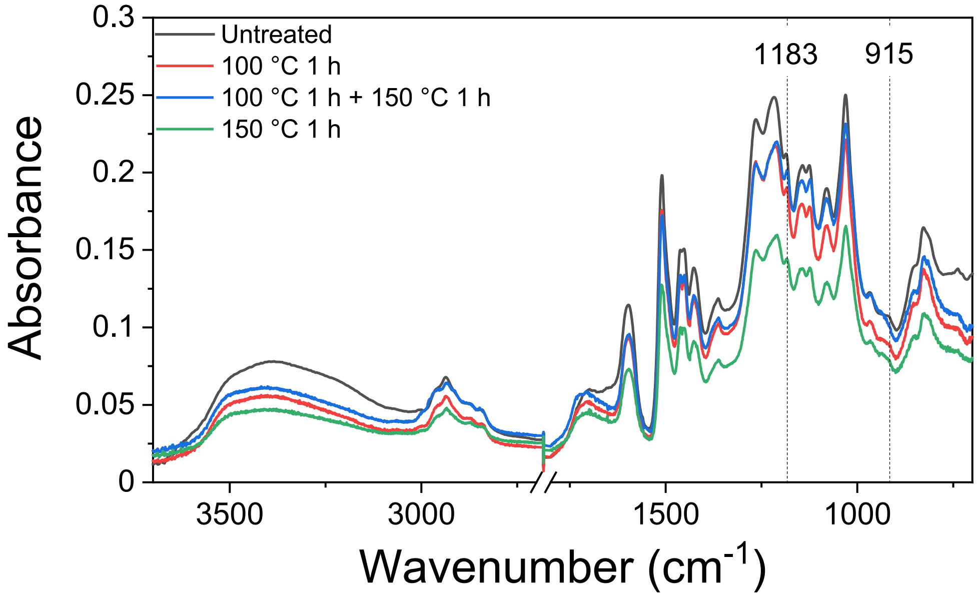

Figure S12. ATR-FTIR spectra of different heat-treated dry hy-LNP10 powders. The dashed lines denote the distinct IR adsorption bands of BADGE at 1183 and 915 cm^{-1}, which are assigned to C-O aromatic ring stretching and C-O stretching of the oxirane group respectively.

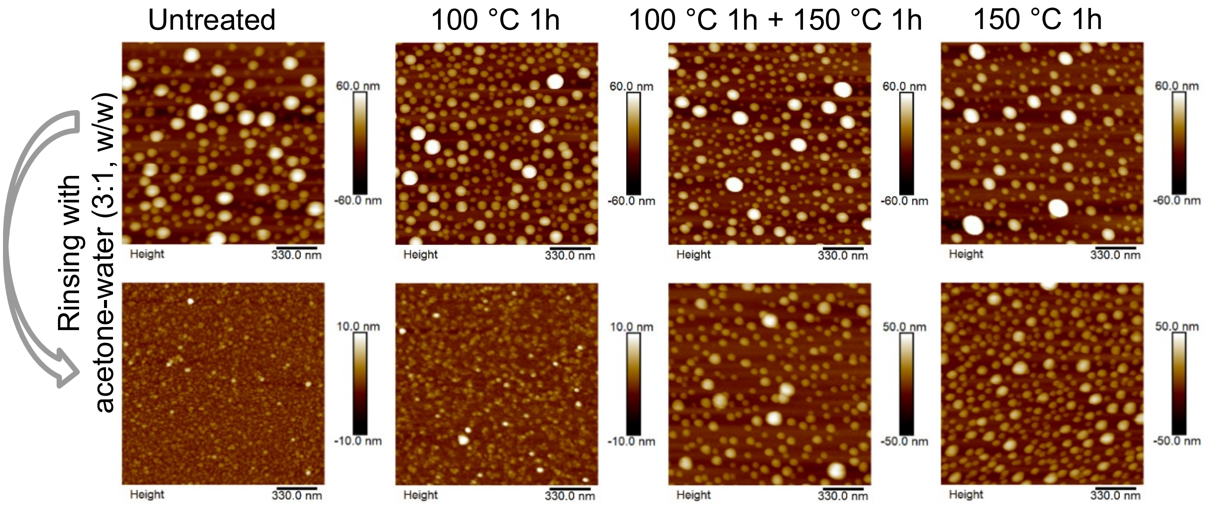

Figure S13. AFM height images of different heat-treated dry hy-LNP20 monolayer, before and after rinsing with acetone-water (3:1, w/w) are shown. Heat-treatment: 1) at 100 °C for 1 hour; 2) at 100 °C for 1 hour, then gradually increasing the temperature to 150 °C and further heating at 150 °C for another hour; 3) at 150 °C for 1 hour.

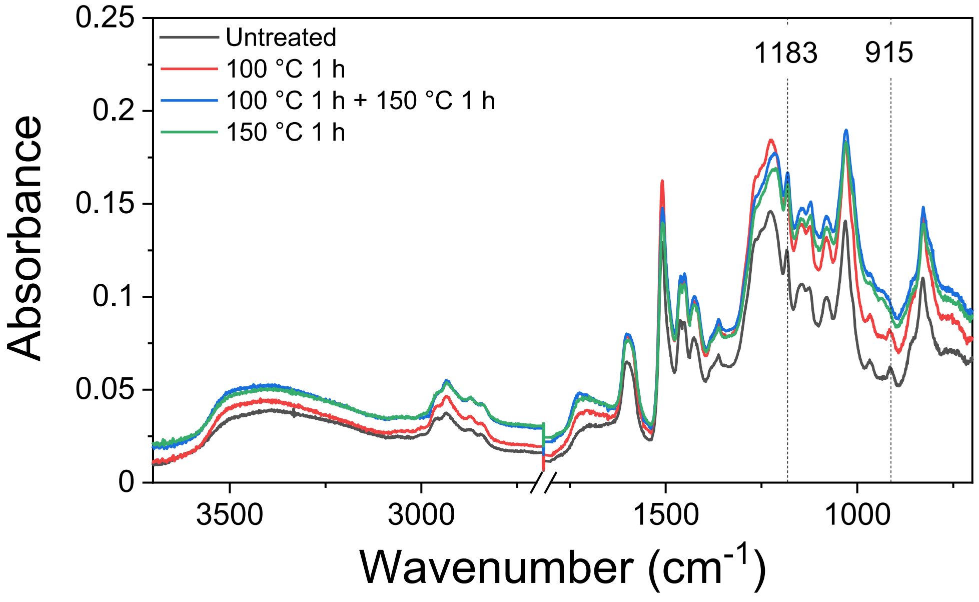

Figure S14. ATR-FTIR spectra of different heat-treated dry hy-LNP20 powders. The dashed lines denote the distinct IR adsorption bands of BADGE at 1183 and 915 cm^{-1}, which are assigned to C-O aromatic ring stretching and C-O stretching of the oxirane group respectively.

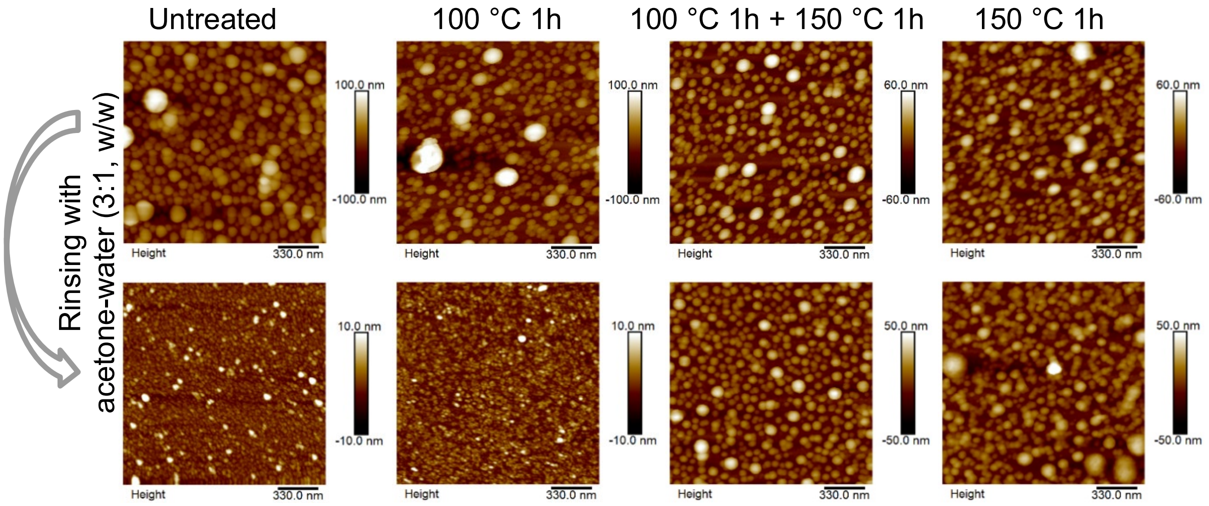

Figure S15. AFM height images of different heat-treated dry hy-LNP30 monolayer, before and after rinsing with acetone-water (3:1, w/w) are shown. Heat-treatment: 1) at 100 °C for 1 hour; 2) at 100 °C for 1 hour, then gradually increasing the temperature to 150 °C and further heating at 150 °C for another hour; 3) at 150 °C for 1 hour.

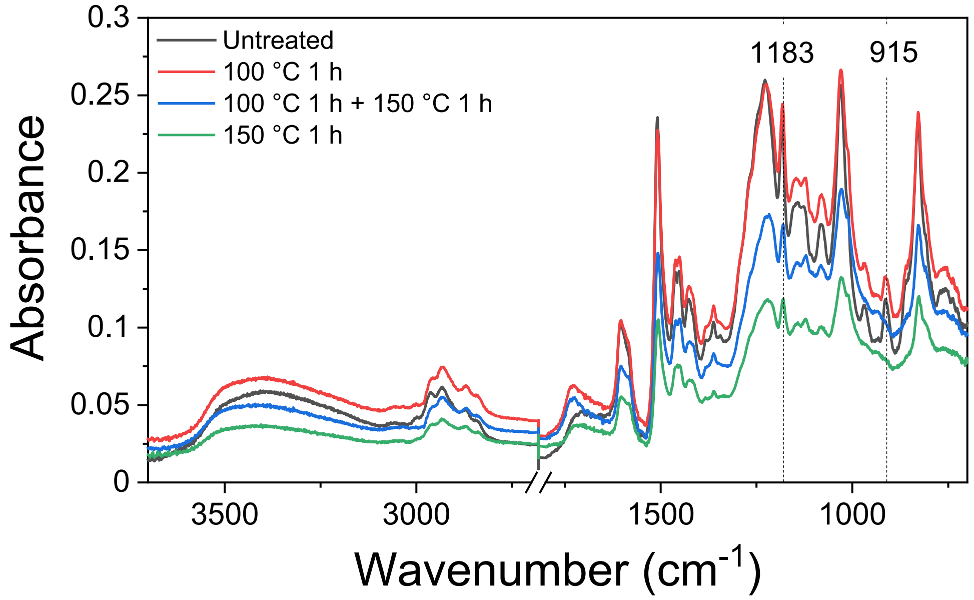

Figure S16. ATR-FTIR spectra of different heat-treated dry hy-LNP30 powders. The dashed lines denote the distinct IR adsorption bands of BADGE at 1183 and 915 cm^{-1}, which are assigned to C-O aromatic ring stretching and C-O stretching of the oxirane group respectively.

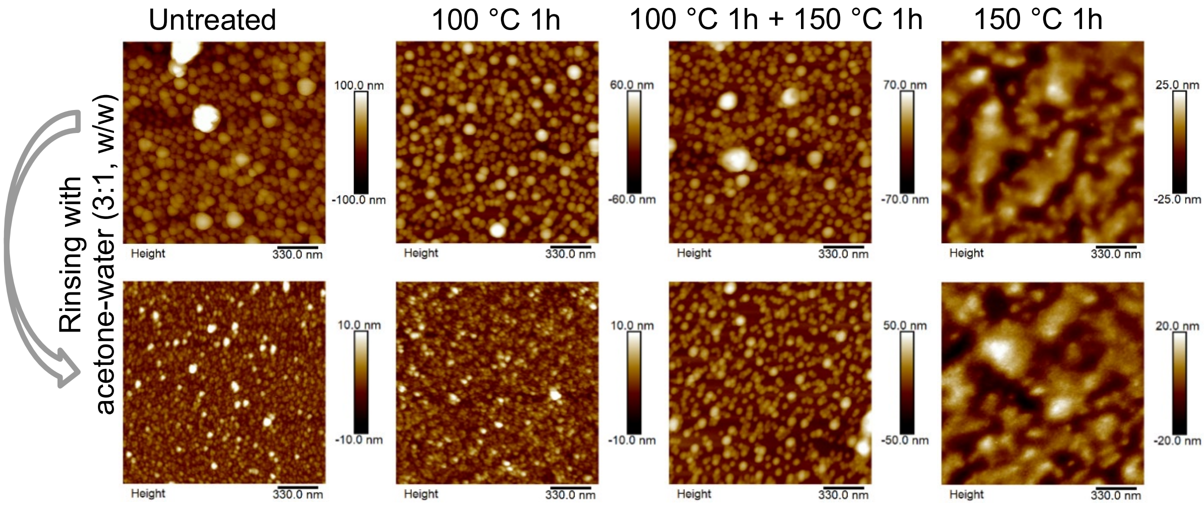

Figure S17. AFM height images of different heat-treated dry hy-LNP40 monolayer, before and after rinsing with acetone-water (3:1, w/w) are shown. Heat-treatment: 1) at 100 °C for 1 hour; 2) at 100 °C for 1 hour, then gradually increasing the temperature to 150 °C and further heating at 150 °C for another hour; 3) at 150 °C for 1 hour.

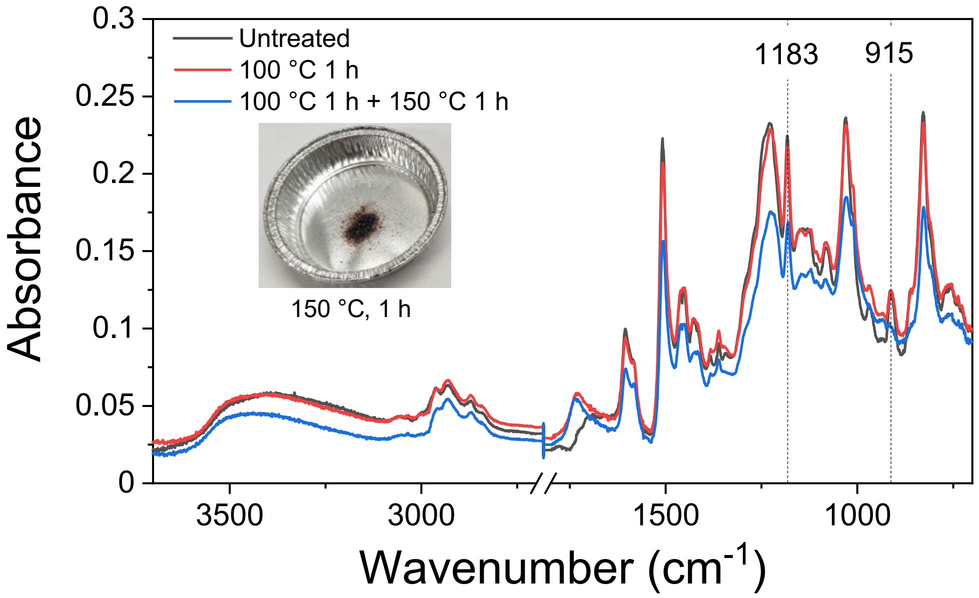

Figure S18. ATR-FTIR spectra of different heat-treated dry hy-LNP40 powders. The dashed lines denote the distinct IR adsorption bands of BADGE at 1183 and 915 cm^{-1}, which are assigned to C-O aromatic ring stretching and C-O stretching of the oxirane group respectively. 150 °C (1 h) treated sample formed a thermoset in the aluminum pan (see the picture in the figure), which was thus not measured with ATR-FTIR.

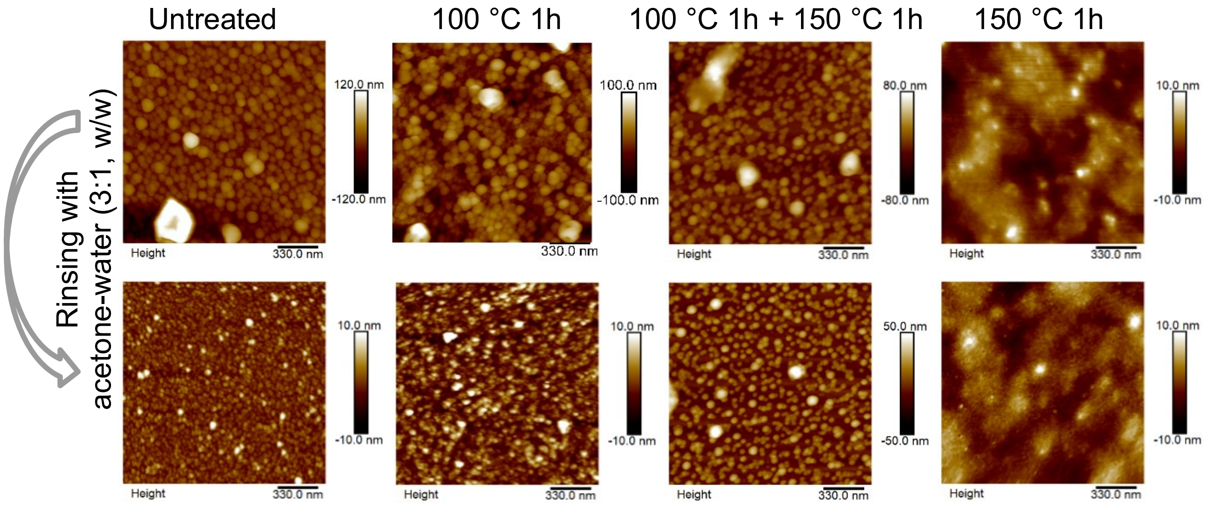

Figure S19. AFM height images of different heat-treated dry hy-LNP50 monolayer, before and after rinsing with acetone-water (3:1, w/w) are shown. Heat-treatment: 1) at 100 °C for 1 hour; 2) at 100 °C for 1 hour, then gradually increasing the temperature to 150 °C and further heating at 150 °C for another hour; 3) at 150 °C for 1 hour.

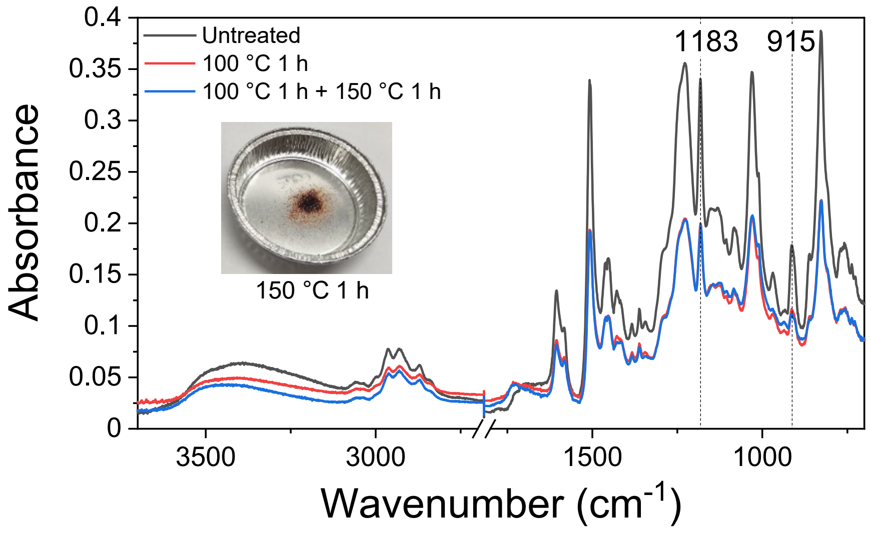

Figure S20. ATR-FTIR spectra of different heat-treated dry hy-LNP50 powders. The dashed lines denote the distinct IR adsorption bands of BADGE at 1183 and 915 cm^{-1}, which are assigned to C-O aromatic ring stretching and C-O stretching of the oxirane group respectively. 150 °C (1 h) treated sample formed a thermoset in the aluminum pan (see the picture in the figure), which was thus not measured with ATR-FTIR.

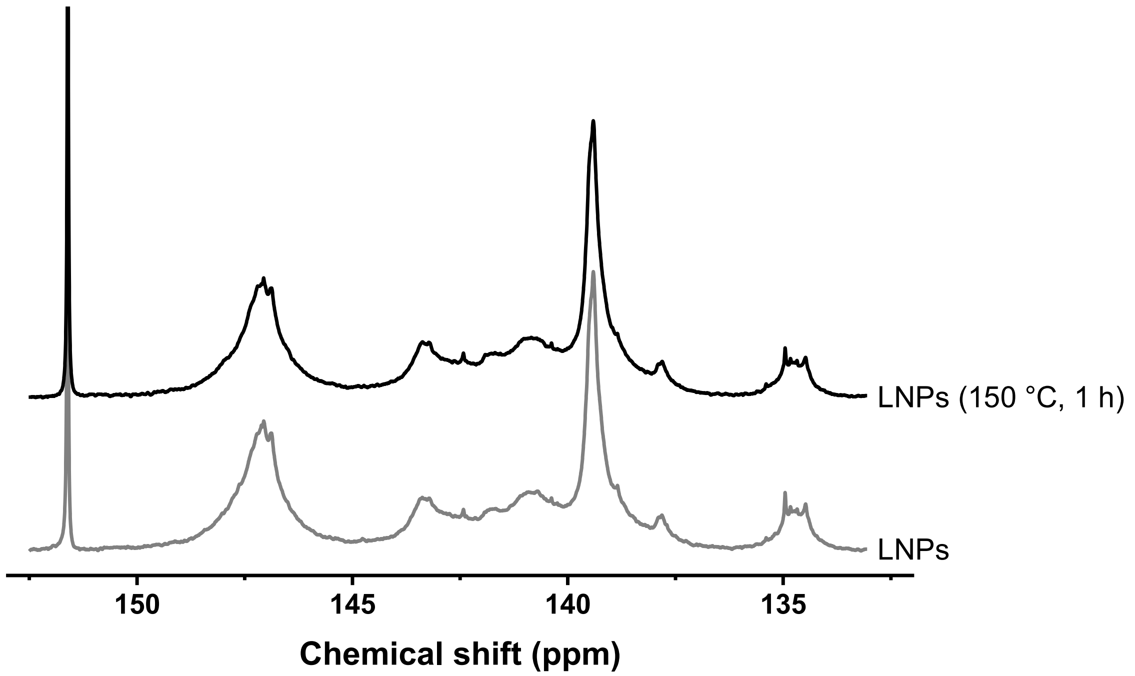

Figure S21. ^{31}P NMR spectra of the untreated and heat-treated (150 ºC, 1 h) LNPs.

Table S3. Concentrations of aliphatic, carboxylic and phenolic OH of the untreated and heat-treated (150 ºC, 1 h) LNPs according to quantitative ^{31}P NMR spectroscopy (unit: mmol/g).

Sample	Aliphatic OH	Carboxylic OH	Phenolic OH	Total OH
LNPs	2.05	0.44	4.07	6.56
LNPs (150 °C, 1 h)	1.95	0.39	4.15	6.50

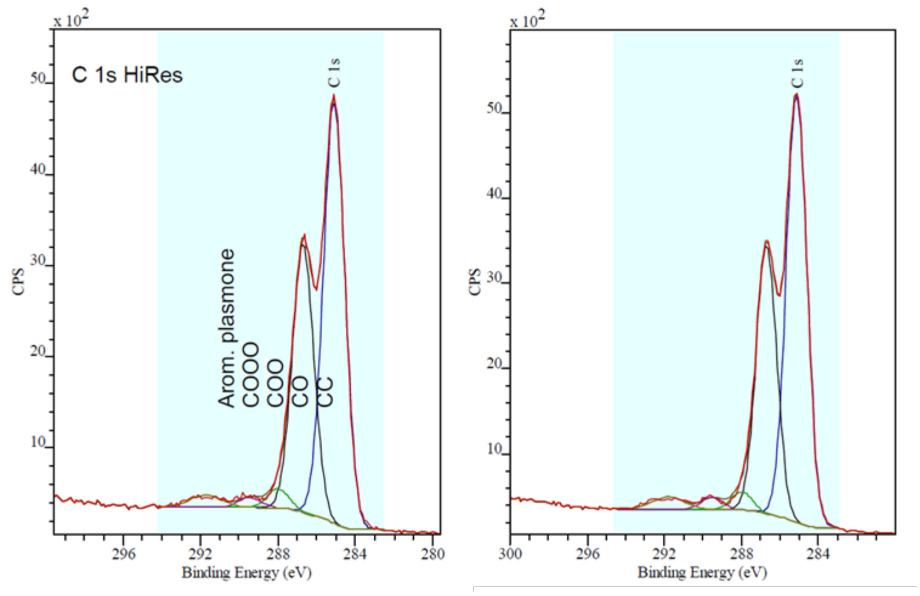

Figure S22. Typical XPS spectra of untreated LNPs (left) and heat-treated (150 °C, 1 h) LNPs (right). The LNP samples were measured with Kratos Analytical AXIS Ultra equipping with DLD detector. High resolution at 286.7 eV was used to obtain the chemical information.

Table S4. Proportions of CC, CO, COO and COOO of the untreated and heat-treated (150 °C, 1 h) LNPs, calculated according to the integrals of decomposed XPS spectra.

	CC	CO	COO	COOO
Untreated (Sample 1)	57.2 %	36.5 %	2.6 %	1.4 %
Untreated (Sample 2)	56.4 %	36.9 %	3.1 %	1.5 %
Untreated (Sample 3)	56.6 %	36.6 %	2.8 %	1.5 %
Heat-treated (Sample 1)	57.4 %	36.0 %	2.5 %	1.6 %
Heat-treated (Sample 2)	57.5 %	36.3 %	2.5 %	1.6 %
Heat-treated (Sample 3)	57.0 %	35.9 %	2.6 %	1.8 %

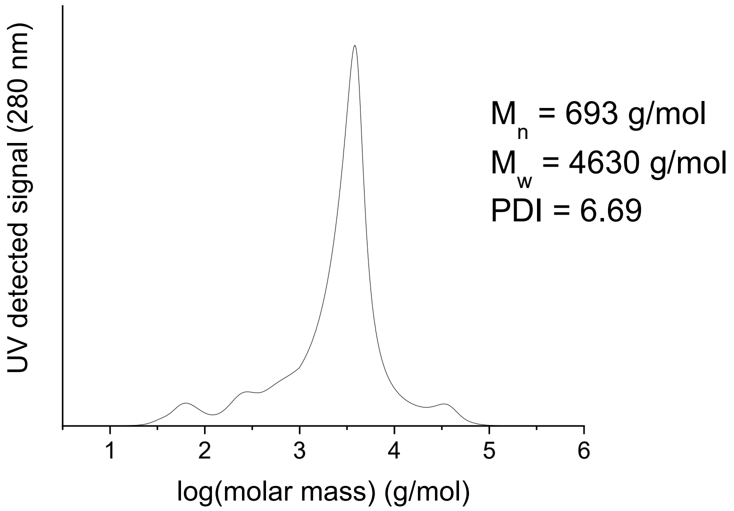

Figure S23. Molar mass distribution of softwood Kraft lignin (SKL) measured with GPC (Agilent Multidetector GPC E419). SKL was dissolved in 0.1 M NaOH at the concentration of 2 mg/ml and filtered through a 0.2 µm syringe filter. A volume of 50 µl was injected into the device with a flow rate of 0.7 ml/min. Polystyrene sulfonate was used as the standard for calibration. The molar mass was calculated based on the UV signal at 280 nm.
